# Hormone correction of dysfunctional metabolic gene expression in stem cell-derived liver tissue

**DOI:** 10.1186/s13287-025-04238-0

**Published:** 2025-03-11

**Authors:** Alvile Kasarinaite, Maria Jimenez Ramos, Mariana Beltran-Sierra, Elena F. Sutherland, Pedro Arede Rei, Make Zhao, Ying Chi, Meryam Beniazza, Andrea Corsinotti, Timothy J. Kendall, Neil C. Henderson, Jonathan A. Fallowfield, Philippa T. K. Saunders, David C. Hay

**Affiliations:** 1 Centre for Regenerative Medicine, Institute for Regeneration and Repair, The University of Edinburgh, Edinburgh BioQuarter, Edinburgh, EH16 4UU UK; 2 Centre for Inflammation Research, Institute for Regeneration and Repair, The University of Edinburgh, Edinburgh BioQuarter, Edinburgh, EH16 4UU UK; 3 Zhejiang University-University of Edinburgh Joint Institute, Zhejiang University, Haining, China; 4 Single-Cell Multi-Omics Facility, Institute for Regeneration and Repair, The University of Edinburgh, Edinburgh BioQuarter, Edinburgh, EH16 4UU UK; 5 Edinburgh Pathology, University of Edinburgh, Edinburgh, UK; 6 MRC Human Genetics Unit, Institute of Genetics and Cancer, University of Edinburgh, Edinburgh, UK; 7 Centre for Reproductive Health, Institute for Regeneration and Repair, The University of Edinburgh, Edinburgh BioQuarter, Edinburgh, EH16 4UU UK

**Keywords:** Liver, MASLD, MASH, Fibrosis, Estrogen, Testosterone, In vitro models, Human, Pluripotent stem cells, Tissue engineering, Transcriptomics, Single nuclei RNA sequencing, Metabolism, Differentiation

## Abstract

The increase in metabolic dysfunction-associated steatotic liver disease (MASLD) and its progression to metabolic dysfunction-associated steatohepatitis (MASH) is a worldwide healthcare challenge. Heterogeneity between men and women in the prevalence and mechanisms of MASLD and MASH is related to differential sex hormone signalling within the liver, and declining hormone levels during aging. In this study we used biochemically characterised pluripotent stem cell derived 3D liver spheres to model the protective effects of testosterone and estrogen signalling on metabolic liver disease ‘in the dish’. We identified sex steroid-dependent changes in gene expression which were protective against metabolic dysfunction, fibrosis, and advanced cirrhosis patterns of gene expression, providing new insight into the pathogenesis of MASLD and MASH, and highlighting new druggable targets. Additionally, we highlight gene targets for which drugs already exist for future translational studies.

**Supplementary Information:**

The online version contains supplementary material available at 10.1186/s13287-025-04238-0.

## Introduction

Metabolic dysfunction-associated steatotic liver disease (MASLD) is the most common chronic liver disease worldwide [1]. In some individuals, MASLD progresses to metabolic dysfunction-associated steatohepatitis (MASH) [2], the inflammatory phase of disease which can lead to liver cirrhosis and hepatocellular carcinoma (HCC). MASLD is sexually dimorphic with a higher prevalence in men compared to women. However, post-menopausal women exhibit a similar risk of MASLD as men [3]. Additionally, men are more likely to develop MASH, fibrosis, and liver-related complications, in particular HCC [4, 5]. Hormone replacement therapy (HRT) has been shown to reduce the risk of developing MASLD in men and women [6, 7]. However, it is not a suitable treatment for all individuals, nor for long-term administration [8, 9]. Therefore, a greater understanding of sex-dependent differences in liver physiology and homeostasis is needed to develop more targeted approaches to treat metabolic liver disease. In these studies we developed sex-specific and scalable human liver tissue models from pluripotent stem cells (PSCs) [10, 11]. Following phenotyping, male and female liver spheres were treated with sex steroids in the presence or absence of a high energy diet. Following this, we profiled their transcriptome and metabolome. The data collected were benchmarked against the clinical multimodal MASLD database, SteatoSITE [12] and their relevance analysed using robust statistical analysis. Importantly, hormone treatment during MASLD had a protective impact on disease progression, regulating genes important in lipogenesis, fibrosis, metabolism and immunity. Although cell-based models have their limitations, such as tissue architecture and systemic interactions to maintain bodily homeostasis, we believe these data provide important information for the field with targets identified for further validation in vitro and in vivo.

## Materials and methods

### Cell line differentiation and characterisation

P106 and H9 cell lines were purchased from WiCell. PSCs were differentiated, characterised, and exposed to lactate, pyruvate, and octanoate (LPO) as previously described [10, 11]. The lines used in the study were metabolically matched to reduce the influence of cell line specific differences on steroid metabolism and transcriptomic readouts. ALT secretion in cell supernatant was measured 48 h post LPO exposure (Assay Core Services, Institute for Regeneration and Repair). Real-time polymerase chain reaction arrays were performed in line with manufacturer’s instructions [13]. 3-hydroxybutyrate, isocitrate and pyruvate were measured using BHB-Glo™, Metabolite-Glo™ and Pyruvate-Glo™ kits (Promega) [14].

### Transcriptomic characterisation

RNA samples were profiled by NanoString nCounter. The data was analysed using NanoString nSolver 4.0 using an Advanced Analysis [15]. Single nuclei isolation from PSC-derived liver spheres and analysis were performed as previously described [16, 17]. Data were benchmarked against SteatoSITE [12].

### Statistics and reproducibility

All statistical analyses were performed using Prism10 (GraphPad). The data were presented as mean ± SD, and a two-tailed Student’s t-test or the Mann–Whitney test, one-way ANOVA test or two-way ANOVA tests were used as indicated in the figure legends. The *P*-value < 0.05 was considered statistically significant. The number of biological replicates is stated in the figure legends.

## Results and discussion

**Fig. 1:**
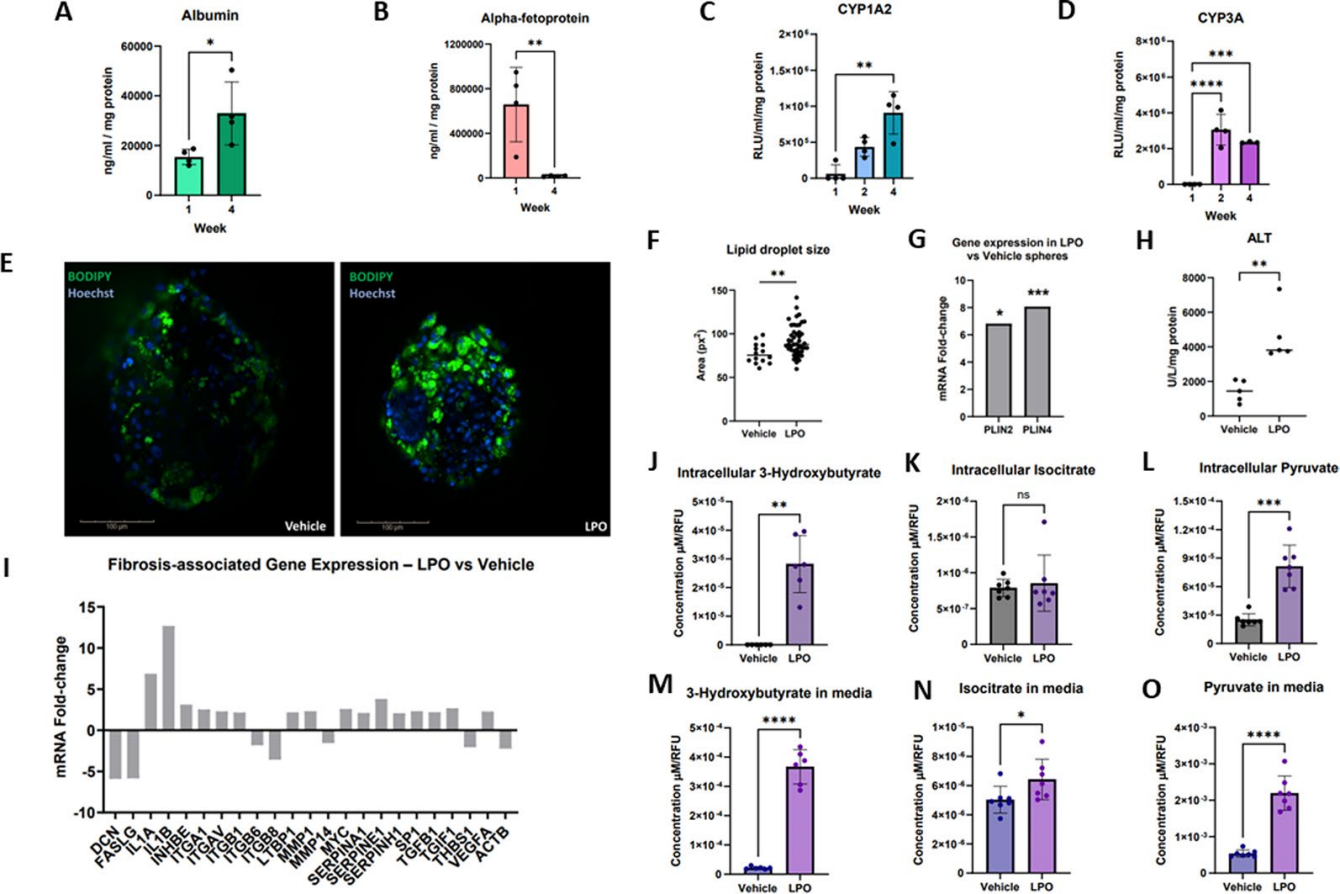
Manufacture and characterisation of liver spheres. **A** Serum albumin was measured in media at 1 and 4 weeks (µg/mL). Thelevels significantly increased at week 4 compared to week 1 (mean ± SD, n = 4), indicating sphere maturation. Culture media was collected after 24 h and assayed using Alpha Diagnostic International ELISA kits as previously described [10]. Data was normalised by total protein content (mg).. The normality of the albumin data was assessed using the Shapiro–Wilk test and an unpaired two-tailed t-test was performed. **B** Alpha-fetoprotein secretion over time. Alpha-fetoprotein levels (µg/mL) were measured at weeks 1 and 4 from liver sphere medium(mean ± SD, n = 4). Culture media was collected after 24 h and quantified using Alpha Diagnostic International ELISA kits as previously described [10]. The normality of the protein data was assessed using the Shapiro–Wilk test and an unpaired two-tailed t-test was performed. **C**, **D** Metabolic activity of liver spheres. (C) Cyp1a2 (blue) and **D** Cyp3a (purple) enzyme activities increased from week 1 to week 4 (mean ± SD, n = 4). Cyp P450 activity was measured using P450-Glo assays (Promega) according to manufacturer's instructions [10, 11]The normality of the Cyp1A2 and Cyp3A activity data was assessed using the Shapiro–Wilk test. One-way way analysis of variance (ANOVA; normal distribution) with Tukey’s multiple comparisons test were performed for both Cyp1A2 and Cyp3A activities study significance. **E** Images of liver spheres treated with lactate, pyruvate and octanoate (LPO) or Vehicle were stained using BODIPY (green) [10, 11]. Liver spheres showed an accumulation of larger lipid droplets in the LPO group. (scale bar 100 µm). Liver spheres within the 96-well Gri3D (SUN Bioscience) Imaging plate were fixed with 4% paraformaldehyde at 4 °C for 30 min as previously described [10]. Following this, the wells were washed with PBS at room temperature and stainedwith BODIPY 493/503 (Cambridge Bioscience LTD). Liver spheres were counterstained with NucBlue Hoechst 33,342 (Sigma-Aldrich) for 30 min at room temperature and prepared for imaging. The spheres were imaged within the Gri3D Imaging plate using an Opera Pheonix using a 40 X water objective. **F** Quantification of lipid droplet size within liver spheres with performed using the Harmony image analysis software (PerkinElmer). The maximum projection analysis of the spot area (px^2^) was assessed using Harmony and visualised in GraphPad Prism v.10. Each data point shows the mean of the droplet area of an individual sphere (mean ± SD). Data was assessed using Shapiro–Wilk normality test and unpaired two-tailed t-test to assess the significance between the groups. **G** Expression of perilipin-2 and 4 (PLIN2 and PLIN4) mRNAs in week 3–4 spheres was significantly increased following LPO treatment. RNAs were extracted using RNeasy MiniPrep (Qiagen) as per manufacturer’s instructions. From the extracted RNA, cDNA was synthesised using RT2 First Strand Kit (Qiagen) for qPCR. Samples were analysed with LightCycler® 480 SW1.5. Data normality was assessed using Shapiro–Wilk normality tests and the significance of P-value was determined using a Mann–Whitney test (mean ± SD, n = 9). **H** Alanine transaminase (ALT) levels (U/L) were analysed following treatment with LPO and Vehiclein week 3–4 liver spheres (mean ± SD, n = 5). Supernatants were collected after 48 h and ALT concentrations were measured and normalised to total protein (mg). Datasets were assessed using Shapiro–Wilk normality test and Mann–Whitney tests. **I** Expression of genes associated with development of fibrosis quantified by real-time qPCR gene array (Qiagen RT2 Profiler PCR array) in week 3 liver spheres with fold-change calculated comparing LPO versus vehicle.. IL1A (> fivefold) and IL1B (> tenfold), INHBE, ITGA1, ITGAV, ITGB1, MMP1, MMP14, SERPINA1, SERPINE1, SERPINH1, SP1, TGFB1, TGIF1, VEGFA (> twofold) were upregulated in LPO spheres compared to the vehicle. DCN and FASLG (> fivefold), ITGB8, THBS1 and ACTB (> twofold) were downregulated in LPO spheres compared to the vehicle after the qPCR analysis (n = 2). (**J**–**L**) The impact of LPO treatment on TCA metabolites intracellular levels of (**J**) 3-hydroxybutyrate, (**K**) isocitrate and (L) pyruvate were measured and compared to control (mean ± SD, n = 6). (**M**–**O**) Secreted levels of (**M**) 3-hydroxybutyrate, (**N**) isocitrate and (**O**) pyruvate were measured (mean ± SD, n = 6). To measure 3-hydroxybutyrate, isocitrate and pyruvate (Promega) intracellular and extracellular levels, the supernatant and the spheres were collected and processed according to the manufacturer’s instructions. Results were normalised for viability using the CellTiter-Fluor.™ cell viability kit (Promega) performed as per the manufacturer’s instructions. Data was compared using Shapiro–Wilk normality tests and the unpaired t-tests to determine the significant differences between the groups. P-values are indicated above the graphs as follows: ns (*P* > 0.05), * (*P* < 0.05), ** (*P* ≤ 0.01), *** (*P* ≤ 0.001) and **** (*P* ≤ 0.0001)

To date there have been limited studies on sex hormone effects on liver pathology [18, 19]. We employed human pluripotent stem cells (PSC) to generate human liver models. They were differentiated and characterised as before [10] [Supplementary Fig. 1]. Stem cell derived liver tissue was composed of hepatic progenitors expressing HNF4α (> 99%), alpha-fetoprotein (AFP) (> 96%), and low levels of albumin (< 3%)] as well as endothelial and stellate cell populations, expressing CD144 (> 94% positive) and PDGFRβ (> 92% positive) respectively [Supplementary Fig. 1]. Hepatocytes, endothelial, and stellate cells were mixed at a ratio of 10:3:1, and allowed to self-assemble in tissue culture microwells. Liver spheres displayed stable and mature phenotype over time confirmed by albumin secretion, CYP1A2 and CYP3A metabolic function in combination with reduced AFP secretion [Supplementary Figs. 2 and 3, Fig. 1A–D].

To induce macrovesicular steatosis, liver spheres were exposed to a cocktail of lactate, pyruvate and octanoate (LPO) [10, 20, 21]. They displayed increased lipid storage and droplet size, expression of perilipin-2 and 4, and leakage of alanine aminotransaminase [Fig. 1E–H]. We also detected increased expression of genes associated with tissue fibrosis, including IL1A (> fivefold) and IL1B (> tenfold), INHBE, ITGA1, ITGAV, ITGB1, MMP1, MMP14, SERPINA1, SERPINE1, SERPINH1, SP1, TGFB1, TGIF1, VEGFA (all > twofold) and decreased DCN and FASLG (> fivefold), and ITGB8, THBS1 and ACTB (> twofold) [Fig. 1I]. Underpinning these changes in gene expression were alterations in cellular bioenergetics with the saturation of fatty acid β-oxidation and overloading of the tricarboxylic acid (TCA cycle) detected [Fig. 1J–O].

To study the effect of hormone signalling, female liver spheres were exposed to a physiological concentration of 17β-estradiol (E2) prior to the induction of steatosis. Gene expression was analysed using NanoString metabolism and fibrosis panels. This was benchmarked against a clinical database of MASLD and MASH (SteatoSITE) [12] (covering stages F0/1 to F4 of disease). Female LPO-treated liver spheres, without E2, displayed a strong correlation with stage F4 samples in mixed and sex stratified analyses (ρ = 0.46, *P* < 0.0001 vs. LPOE, ρ = 0.41, *P* < 0.0001) and (ρ = 0.34, *P* < 0.001 vs ρ = 0.32, *P* < 0.01) respectively [Supplementary Figs. 4 and 5, Supplementary Tables 1, 2, 3 and 4]. Metabolically equivalent male liver spheres were exposed to a physiological concentration of 10^–8^ M testosterone (T) prior to the induction of steatosis [Supplementary Fig. 6, 7 and 8, Supplementary Tables 5, 6, 7 and 8]. Despite improvements in male spheres following hormone treatment, the addition of T did not correct the late stage of fibrotic gene expression (F4) to the same extent as in E2 pre-treated female spheres.

**Fig. 2:**
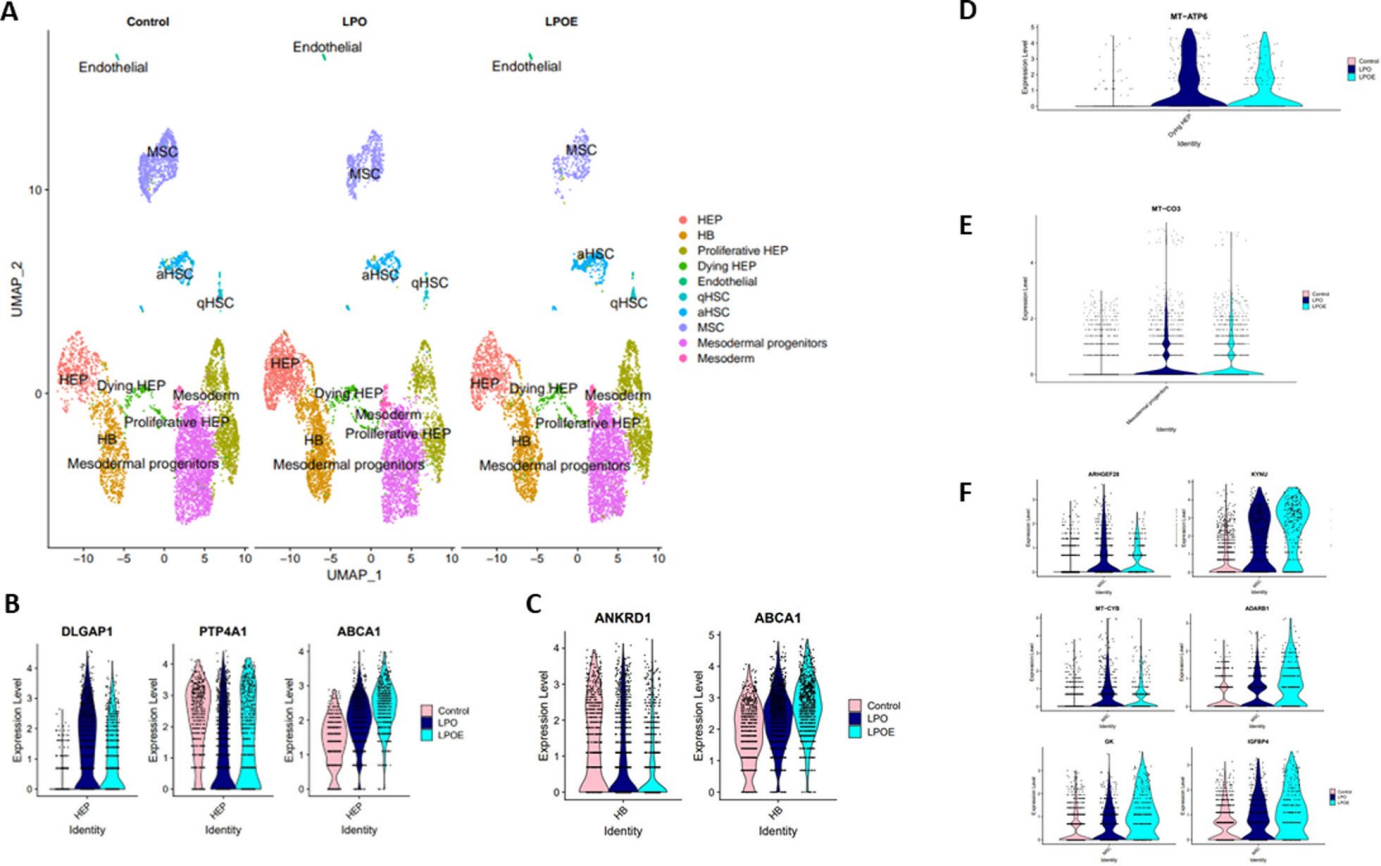
Single nuclei RNA-sequencing of female spheres. H9-derived (female) liver spheres were treated with 10^–8^ M 17β-estradiol  (E2) prior to the induction of steatosis and compared to non-treated control spheres. 3 × micromolds, containing 256 spheres each, were pooled together, with 2 replicates per condition. The conditions included control, LPO and LPO with E2 (LPOE) (n = 2; 1 × n = 768 spheres). Single nuclei isolation was performed as previously described [16]. The resuspended pellet was stained with 7AAD (1:100) and incubated on ice for 5–10 min for FACS nuclei sorting using BD FACSAriaTM II. All 7AAD + nuclei were sorted into 500 μL PBS/0.2% BSA, aiming for 100,000 – 200,000 nuclei per sample. The nuclei were spun down at 1,000 rcf for 5 min and counted after FACS using Trypan-Blue for Haemocytometer and Cell Count for Evios. After sorting and counting, approximately 20,000 nuclei per sample were processed for single-nuclei sequencing using 10X Genomics 3' V3.1 reagents and following manufacturer instructions. Libraries were quantified using an Agilent Technologies TapeStation 2200 (Agilent) and sent for sequencing on Illumina P3 and S1 flow cells. Sequenced replicates were grouped into three datasets (control, LPO and LPOE) for computational analysis. Sequenced raw data was processed using CellRanger v.7.1.0 and CellRanger count pipeline. Downstream analyses were performed in R (v.4.3.0) using Seurat package (v.4.3.0.) following the Seurat pipeline (https://satijalab.org/seurat/articles/pbmc3k_tutorial.html) recommendations. **A** UMAP dimensionality reduction analysis of snRNA-seq data from control, LPO and LPOE treatments. The conserved genes between the datasets (control, LPO and LPOE) were assessed and top genes were compared to signatures available in the literature. In addition, the cell-specific gene marker expressions from the literature were investigated. 12 separate clusters were identified with 0.3 resolution within all the datasets, including hepatocyte (HEP), proliferative hepatocyte (Proliferative HEP) and dying hepatocyte (Dying HEP), hepatic progenitor (HB), endothelial, active hepatic stellate (aHSC) and quiescent hepatic stellate (qHSC), mesenchymal stroma (MSC), mesodermal progenitors and other mesodermal cells (Mesoderm). **B** Violin plots showing the expression of the most differentially expressed hepatocyte genes between LPO (compared to control) and LPOE (compared to control) (|log_2_FC(LPO)-(log_2_FC(LPOE)|≥ 0.5, adjusted P-value < 0.05 according to Bonferroni correction). The same statistical analysis applies to all the following plots. The plots show the expression of DLGAP1, PTP4A1 and ABCA1 in all datasets. **C** Violin plots showing the expression of the most differentially expressed hepatic progenitor  (HB) genes between LPO (compared to control) and LPOE (compared to control). The plots show the expression of ANKRD1 and ABCA1 in all datasets. **D** Violin Plot showing the expression of the most differentially expressed dying hepatocyte genes between LPO (compared to control) and LPOE (compared to control). The plots show the expression of MT-ATP6 in all datasets. **E** Violin plots showing the expression of the most differentially expressed mesodermal progenitor genes between LPO (compared to control) and LPOE (compared to control). The plots show the expression of MT-CO3 in all datasets. **F** Violin plots showing the expression of the most differentially expressed mesenchymal stromal cell (MSC) genes between LPO (compared to control) and LPOE (compared to control). The plots show the expression of GK, KYNU, ADARB1, IGFBP4, ARHGEF28 and MT-CYB in all datasets. All selected differentially expressed genes from female spheres were compared with SteatoSITE gender-specific RNA-sequencing gene signatures (adjusted P-value < 0.05 according to Benjamini–Hochberg procedure) for F4 fibrosis stage and were presented in this study

To better understand the effects of hormone signalling at the cellular level, we employed single-nuclei RNA-sequencing (snRNA-seq). Female liver spheres were composed of hepatocytes (HEP), hepatic progenitors (HB), proliferative hepatocytes (Proliferative HEP), dying hepatocytes (Dying HEP), endothelial cells, mesodermal progenitors, mesenchymal stromal cells (MSC), quiescent (qHSC) and activated (aHSC) hepatic stellate cells [Fig. 2A and Supplementary Fig. 9, Supplementary Table 9]. Following exposure to LPO, we observed an increased number of cells within HB, HEP and MSC clusters [Fig. 2 and Supplementary Fig. 9, Supplementary Table 9]. However, following E2 treatment there were more cells within the Proliferative HEP cluster expressing cancer markers, highlighting the risk of hormone-based therapies [Fig. 2A and Supplementary Fig. 9, Supplementary Table 9]. Additionally, DLGAP1 was downregulated (> 1.5-fold), whilst PTP4A1 and ABCA1 were upregulated (> 1.45-fold) in HEP cluster [Fig. 2B, Supplementary Tables 10 and 11]. In E2 treated hepatic progenitors the disease marker, ANKRD1, was downregulated (> 1.5-fold) whereas the fat metabolism marker, ABCA1, was upregulated (> 1.45-fold) [Fig. 2C, Supplementary Tables 12 and 13]. Interestingly, we detected a separate cluster for Dying HEPs, where MT-ATP6 was downregulated (> 1.45-fold) after E2 treatment [Fig. 2D, Supplementary Tables 14 and 15]. In mesodermal progenitors we observed a downregulation of the autophagy markers MT-CO3 (> 1.4-fold) following E2 treatment [Fig. 2E, Supplementary Tables 16 and 17]. In the MSC cluster, GK, KYNU, and ADARB1 and IGFBP4 were upregulated (> 1.5-fold), whilst ARHGEF28 (> 1.5-fold) and MT-CYB (> 1.4-fold) were downregulated following E2 treatment [Fig. 2F, Supplementary Tables 18 and 19].

**Fig. 3:**
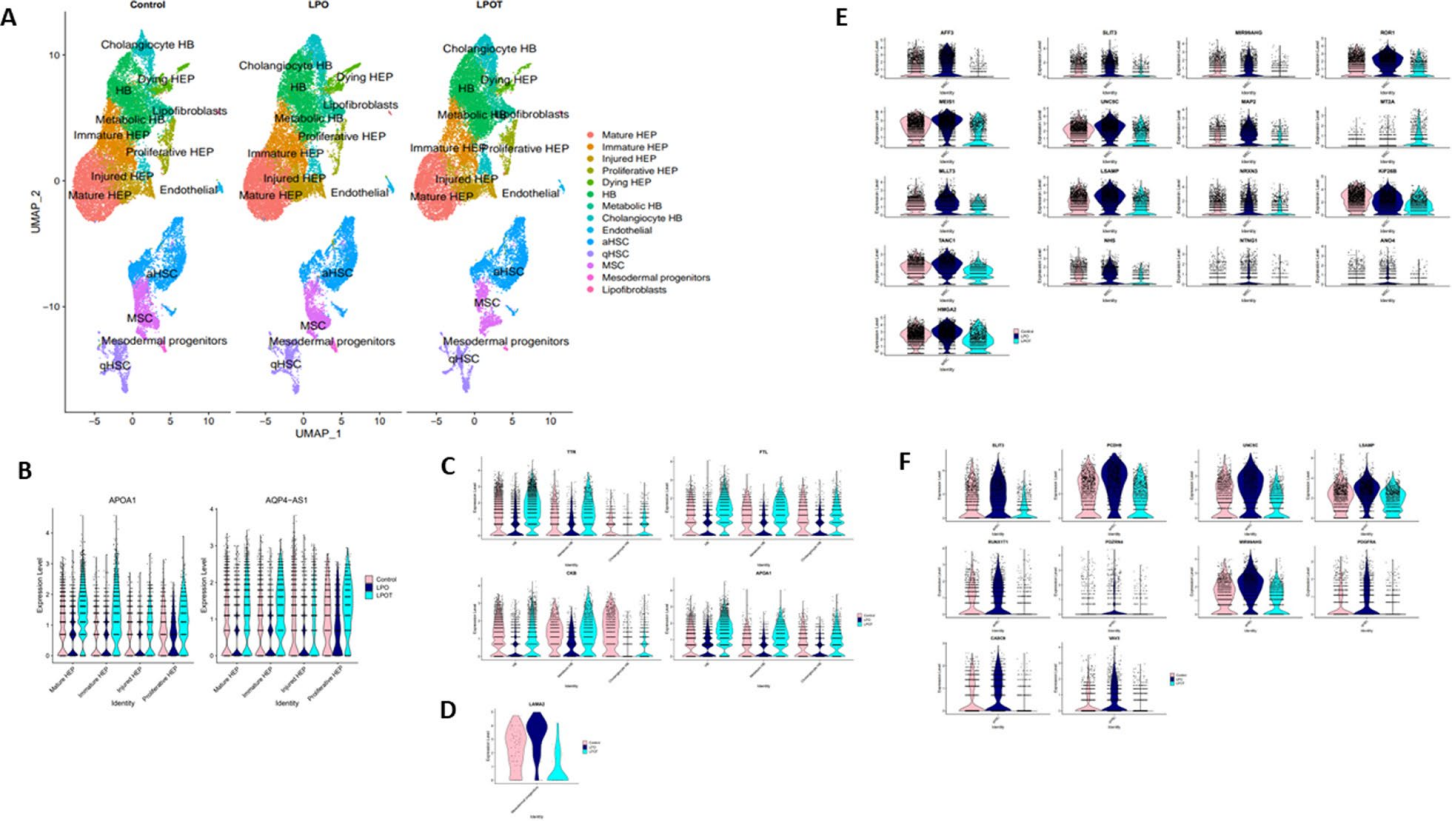
Single nuclei RNA-sequencing of male spheres. P106-derived (male) liver spheres were treated with 10^–8^ M testosterone (T) prior to the induction of steatosis and compared to non-treated control spheres. 3 × micromolds, containing 256 spheres each, were pooled together, with 3 replicates per condition. The conditions included control, LPO and LPO with T (LPOT) (n = 3; 1 × n = 768 spheres). For single nuclei isolation – TST method previously described in [16] was chosen. The samples were processed and sequenced as described in Fig. 2 legend. Sequenced replicates were grouped into three datasets (control, LPO and LPOT) for computational analysis. **A** UMAP dimensionality reduction analysis of snRNA-seq data from control, LPO and LPOT treatments. 14 separate clusters were identified with 0.3 resolution within all the datasets, including hepatocyte (Mature HEP, Immature HEP, Injured HEP, Proliferative HEP and Dying HEP), hepatic progenitor (HB, Metabolic HB and Cholangiocyte HB), endothelial, hepatic stellate (aHSC and qHSC), mesenchymal stroma (MSC), mesodermal progenitors and lipofibroblasts. **B** Violin plots showing the expression of the most differentially expressed hepatocyte genes between LPO (compared to control) and LPOT (compared to control) (|log_2_FC(LPO)—(log_2_FC(LPOT)|≥ 1, adjusted P-value < 0.05 according to Bonferroni correction). The same statistical analysis was used for all the following plots. The plots show the expression of APOA1 and AQP4-AS1 in all datasets. **C** Violin plots showing the expression of the most differentially expressed hepatic progenitor genes between LPO (compared to control) and LPOT (compared to control). The plots show the expression of CKB, FTL, TTR and APOA1 in all datasets. **D** Violin Plot showing the expression of the most differentially expressed mesodermal progenitor genes between LPO (compared to control) and LPOT (compared to control). The plots show the expression of LAMA2 in all datasets. **E** Violin plots showing the expression of the most differentially expressed mesenchymal stromal cell (MSC) genes between LPO (compared to control) and LPOT (compared to control). The plots show the expression of AFF3, SLIT3, MIR99AHG, ROR1, MEIS1, UNC5C, MAP2, MT2A, MLLT3, LSAMP, NRXN3, KIF26B, TANC1, NHS, NTNG1, ANO4 and HMGA2 in all datasets. (**F**) Violin plots showing the expression of the most differentially expressed quiescent stellate cell (qHSC) genes between LPO (compared to control) and LPOT (compared to control). The plots show the expression of SLIT3, PCDH9, UNC5C, LSAMP, RUNX1T1, PDZRN4, MIR99AHG, PDGFRA, CASC9 and VAV3 in all datasets. All selected differentially expressed genes from male spheres were compared with SteatoSITE gender-specific RNA-sequencing gene signatures (adjusted *P*-value < 0.05 according to Benjamini–Hochberg procedure) for F4 fibrosis stage and were presented in this study

Male liver spheres were comprised of hepatocytes (HEP), including mature hepatocytes (Mature HEP), immature hepatocytes (Immature HEP), injured hepatocytes (Injured HEP), proliferative hepatocytes (Proliferative HEP) and dying hepatocytes (Dying HEP); hepatic progenitors (HB), including also cholangiocyte hepatic progenitors (Cholangiocyte HB) and metabolic hepatic progenitors (Metabolic HB); endothelial cells, mesodermal progenitors, lipofibroblasts, mesenchymal stromal cells (MSC), quiescent (qHSC) and activated (aHSC) hepatic stellate cells [Fig. 3A and Supplementary Fig. 10, Supplementary Table 20]. LPO treated liver spheres exhibited an increased number of cells within Mature HEP, Injured HEP, and Dying HEP clusters compared to T treated cells [Fig. 3A and Supplementary Fig. 10, Supplementary Table 20]. Additionally, T treated liver spheres possessed reduced  numbers of Immature HEP and MSCs; and greater amounts of Proliferative HEP, HB, Metabolic HB and Cholangiocyte HB clusters [Fig. 3A and Supplementary Fig. 10, Supplementary Table 20]. Differentially expressed genes in hormone treated cells included, APOA1 and AQP4-AS1 upregulation (> twofold) in hepatocytes, and CKB, FTL, TTR and APOA1 (> twofold) in hepatic progenitors [Fig. 3B and C, Supplementary Tables 21, 22, 23 and 24]. Notably, LAMA2 (> sevenfold) was downregulated in hormone-treated mesodermal progenitors [Fig. 3D, Supplementary Tables 25 and Tables 26]. Downregulated genes in the MSC cluster included AFF3 (> sixfold), SLIT3 (> threefold), MIR99AHG, ROR1, MEIS1, UNC5C, MAP2, MT2A, MLLT3, LSAMP, NRXN3, KIF26B, TANC1, NHS, NTNG1, ANO4 and HMGA2 (> twofold) [Fig. 3E, Supplementary Tables 27 and 28]. Whereas, HSC markers; SLIT3 (> 4.5-fold), PCDH9 (> threefold), UNC5C, LSAMP, RUNX1T1, PDZRN4, MIR99AHG, PDGFRA, CASC9 and VAV3 (> twofold) were downregulated in post T treatment [Fig. 3F, Supplementary Tables 29 and 30].

**Fig. 4:**
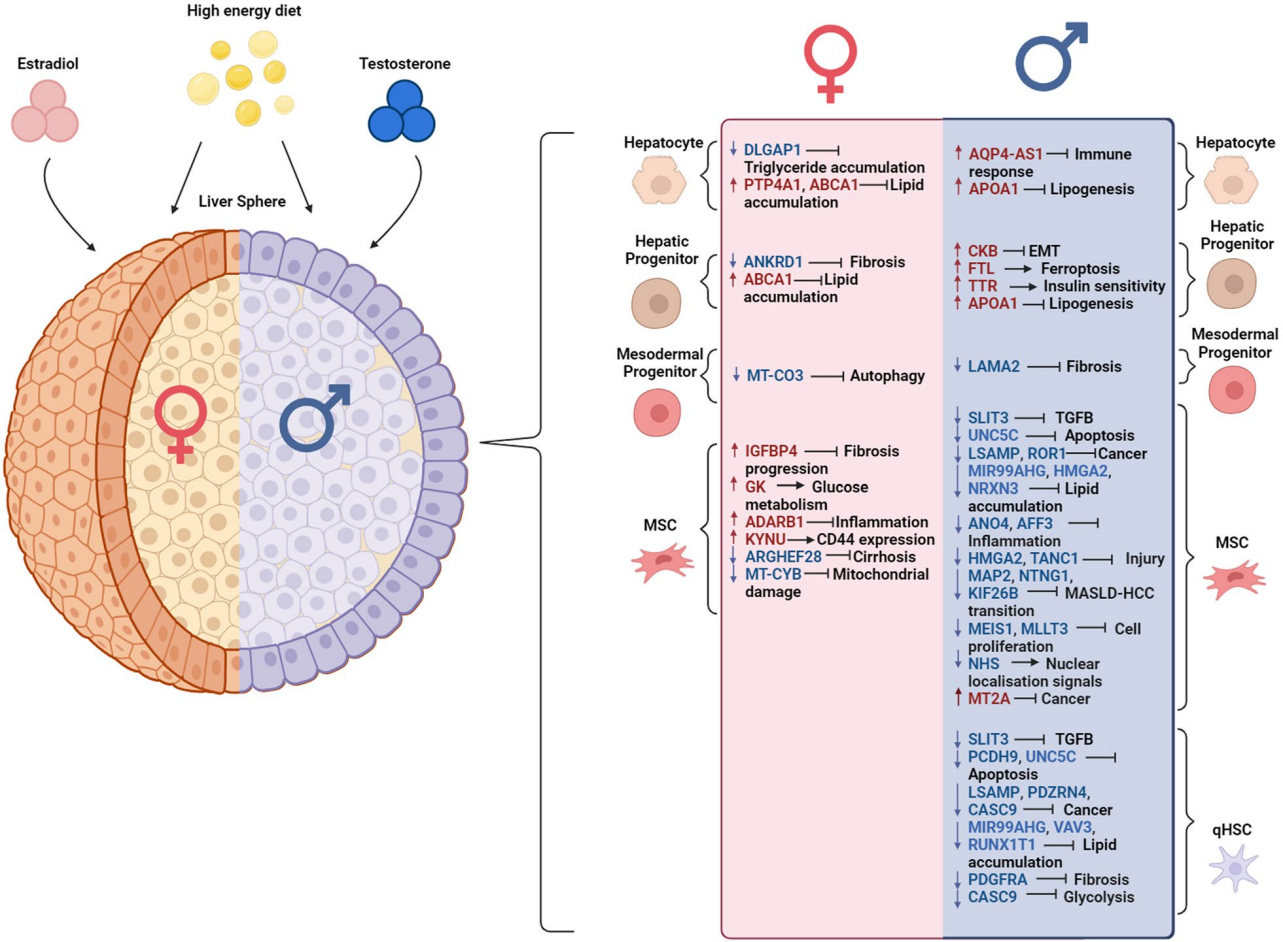
Summary of the impact of sex steroids on gene expression patterns in human liver spheres during MASH modelling. The schematic representation shows the gene expression changes in liver spheres with the hormone treatment prior to the induction of MASH using LPO. Female liver spheres (orange) with E2 (pink) treatment expressed genes involved in protection against late-stage fibrosis in women (red text box), with the red arrows and genes being upregulated in LPOE treatment compared to the LPO treatment; and blue arrows and genes showing the downregulation in LPOE compared to the LPO treatment. Likewise, male liver spheres (blue) expressed genes associated with protection against late-stage fibrosis in men (blue box), where the red arrows and genes being upregulated in LPOT treatment compared to the LPO treatment; and blue arrows and genes showing the downregulation in LPOT compared to the LPO treatment. Single cell input in disease protection is showed on the right-side of the figure with the pink panel indicating gene expression changes in females following the E2 treatment and the blue panel indicating the gene expression changes in male liver spheres following the T treatment

Taken together, E2 treatment demonstrated protection against advanced cirrhosis (F4) with increased gene expression in female liver spheres with genes, associated with ethanol metabolism [22], ureagenesis [23], sugar metabolism, lipogenesis [24–26], drug metabolism [27], immune response [28–30] and fibrosis [31]. Stratification by sex identified further genes involved in MASH including glucose metabolism and insulin sensitivity [32], ureagenesis [33] and lipid formation [34]. We also discovered gene expression differences to that in the literature. Previously, CYP8B1, PKLR, and SLC2A2 were described as upregulated in human and murine MASLD [24, 35, 36]. The differences in our datasets, supported by SteatoSITE, are likely due to those previous studies not being stratified by sex or were tested  in male only experimental systems [35, 36]. As detailed, E2 treatment had a larger protective effect against late-stage MASH gene expression than T. E2 treatment showed protective effects on hepatocytes and hepatic progenitors against disease development with downregulated expression of DLGAP1 and ANKRD, and increased in PTP4A1 and ABCA1 expression [37–40]. In the mesodermal progenitor cluster, MT-CO3 downregulation was observed suggesting reduced autophagy [41]. Similarly, E2 treatment resulted in increased expression of genes associated with glucose (GK) [42], and tryptophan metabolism (KYNU) genes [43] in MSCs. Whereas, markers of inflammation (ADARB1) [44], mitochondrial damage (MT-CYB) [45], and cirrhosis (ARHGEF28) gene expression were reduced [46]. In male liver spheres, T addition led to increased APOA1 expression within hepatocytes and hepatic progenitors, suggesting improved lipid homeostasis [40]. We also observed increased AQP4-AS1 expression which could be associated with repression of the immune system [47]. T treatment of hepatic progenitors also reduced EMT marker expression (CKB) [48], whilst maintaining a marker of insulin sensitivity (TTR) [49]. Additionally, we detected reduced ferritin light chain (FTL) expression, which is commonly upregulated gene in MASH patients [50]. In mesodermal progenitors, T downregulated the expression of LAMA2 which is associated with late-stage fibrosis in patients [51]. Additionally, T displayed a protective effect in MSCs by inhibiting TGFβ signalling (SLIT3) [52], repressing apoptosis (UN5C) [53] and cancer development (ROR1, MT2A) [54, 55], and reducing lipid accumulation (HMGA2, MIR99AHG and NRNX3) [56–58]. T also downregulated the expression of genes associated with inflammation and the transition from MASLD to HCC (ANO4; HMGA2; MAP2, NTNG1 and KIF26B; MEIS1 and MLLT3; and NHS) [56, 59–65]. Interestingly, LSAMP, a HCC suppressor [66], AFF3 an immune regulator [67] and TANC1 a liver injury marker [68] were significantly downregulated following T pre-treatment. This was in contrast to the data reported in the literature. More generally, T-treated liver spheres displayed downregulation of genes associated with apoptosis (PCDH9) [69], cancer progression (CASC9 and LSAMP) [66, 70] and lipid accumulation (VAV3) [71]. Furthermore, fibrosis (PDGFRα) [17] and glycolysis (CASC9) related gene expression were reduced following hormone treatment. Although sex hormones were protective against development of MASH [summarised in Fig. 4], the gene signatures differed between the sexes. Notably, there are approved drugs (https://go.drugbank.com/) [72] for the genes addressing the impaired pathways in MASLD/MASH, such as hepatocyte metabolism (ADH1C and CPS1) [22, 23], insulin sensitivity and glucose metabolism (PKLR, FTL and TTR) [36, 49, 50], lipid accumulation (ABCA1) [39], MASLD to cancer transition (MT2A and MAP2) [55, 60], and fibrosis and scarring (CKB, PDGFRA) [48, 69]. Furthermore, the delivery of these drugs could be optimised using various strategies, including the use of nanomaterials [73], in future translational studies.

In conclusion, our research identified sex differences in MASLD and MASH progression, with E2 and T playing a protective role against end stage disease. In particular, we highlight new patterns of sex specific gene expression with several of those targets already druggable. We believe our research highlights the importance of sex-specific based modelling in human biomedical research.

## Supplementary Information

Additional file 1

Additional file 2

Additional file 3

Additional file 4

Additional file 5

Additional file 6

Additional file 7

Additional file 8

Additional file 9

Additional file 10

Additional file 11

Additional file 12

Additional file 13

Additional file 14

Additional file 15

Additional file 16

Additional file 17

Additional file 18

Additional file 19

Additional file 20

Additional file 21

Additional file 22

Additional file 23

Additional file 24

Additional file 25

Additional file 26

Additional file 27

Additional file 28

Additional file 29

Additional file 30

Additional file 31

Additional file 32

Additional file 33

Additional file 34

Additional file 35

Additional file 36

Additional file 37

Additional file 38

Additional file 39

Additional file 40

Additional file 41

## Data Availability

Provided in supplementary information.
