# Supplementary figures and images for: Hormone correction of dysfunctional metabolic gene expression in stem cell-derived liver tissue
Source: Stem Cell Res Ther. 2025 Mar 11;16:130. doi: 10.1186/s13287-025-04238-0 (PMC11899078; doi:10.1186/s13287-025-04238-0)

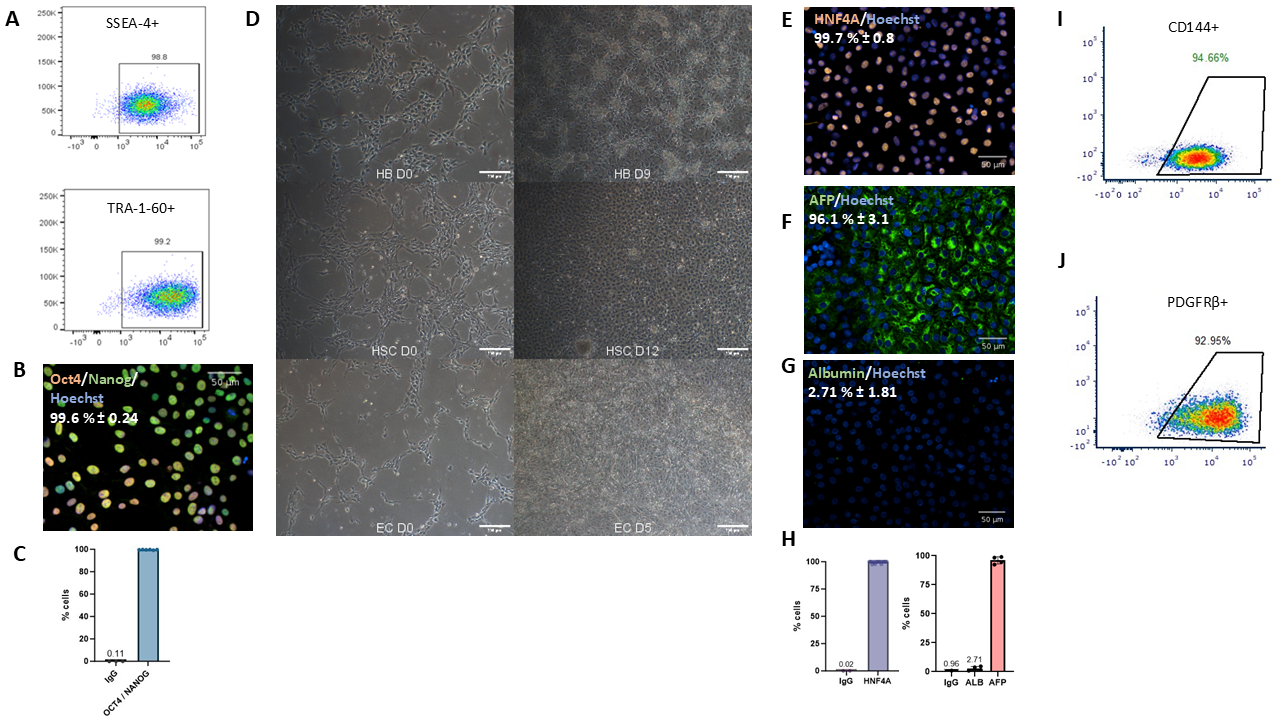

Supplement: Supplementary file 1 — Additional file 1 [file 13287_2025_4238_MOESM1_ESM.tif]

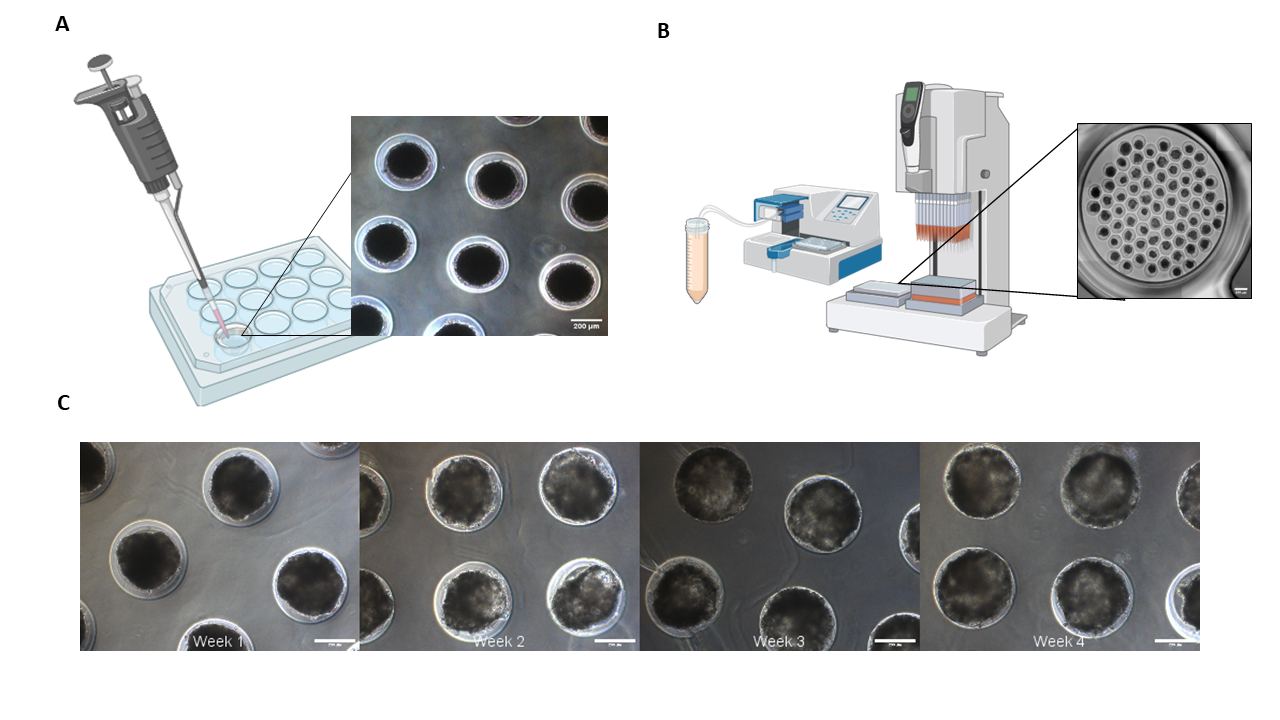

Supplement: Supplementary file 2 — Additional file 2 [file 13287_2025_4238_MOESM2_ESM.tif]

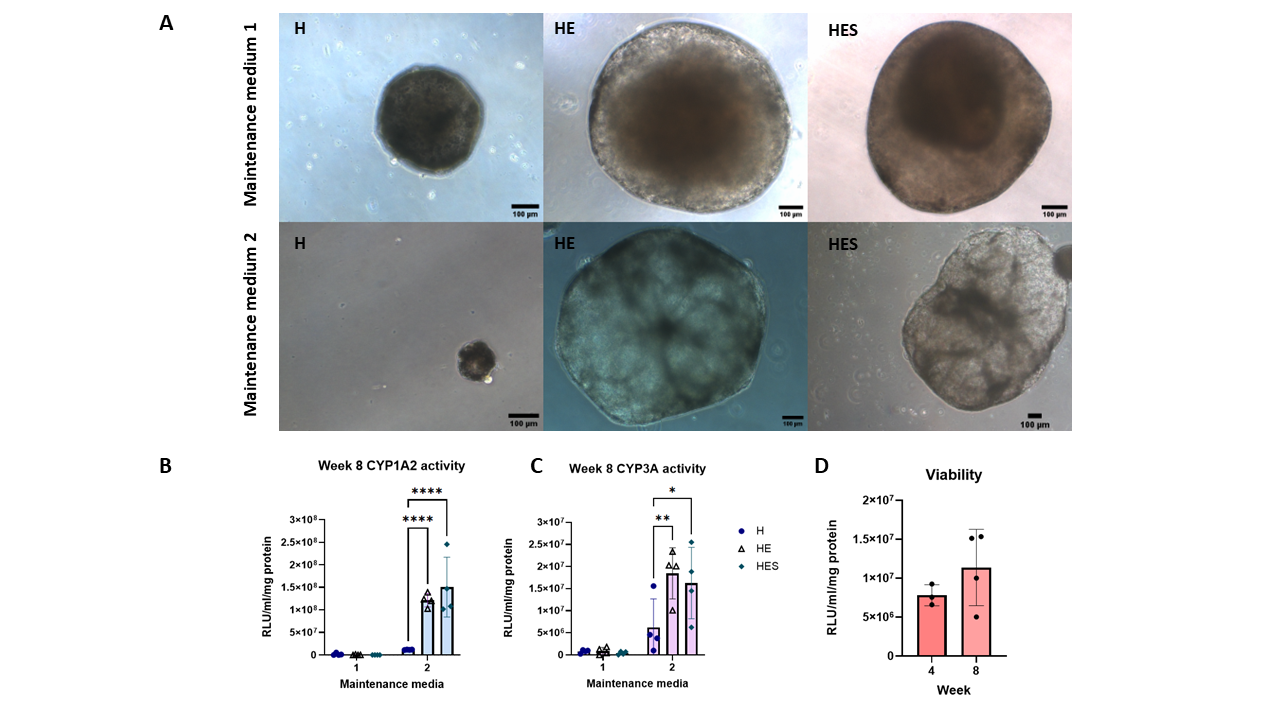

Supplement: Supplementary file 3 — Additional file 3 [file 13287_2025_4238_MOESM3_ESM.tif]

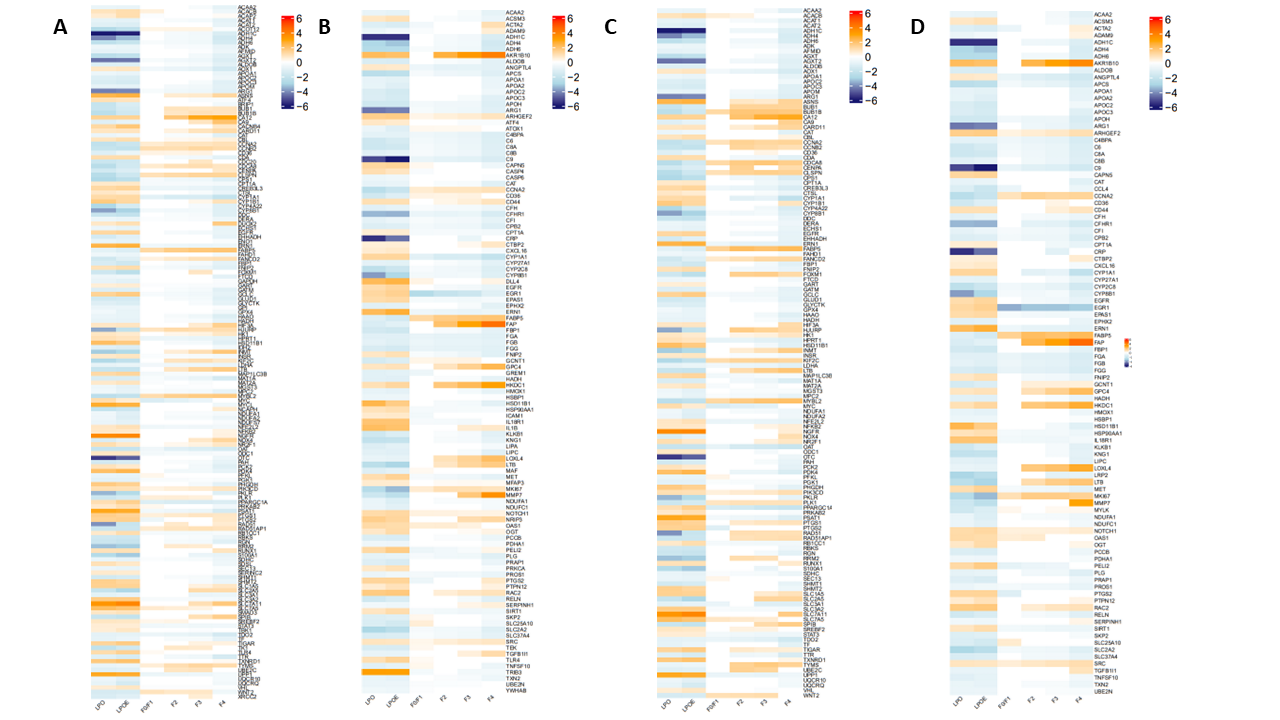

Supplement: Supplementary file 4 — Additional file 4 [file 13287_2025_4238_MOESM4_ESM.tif]

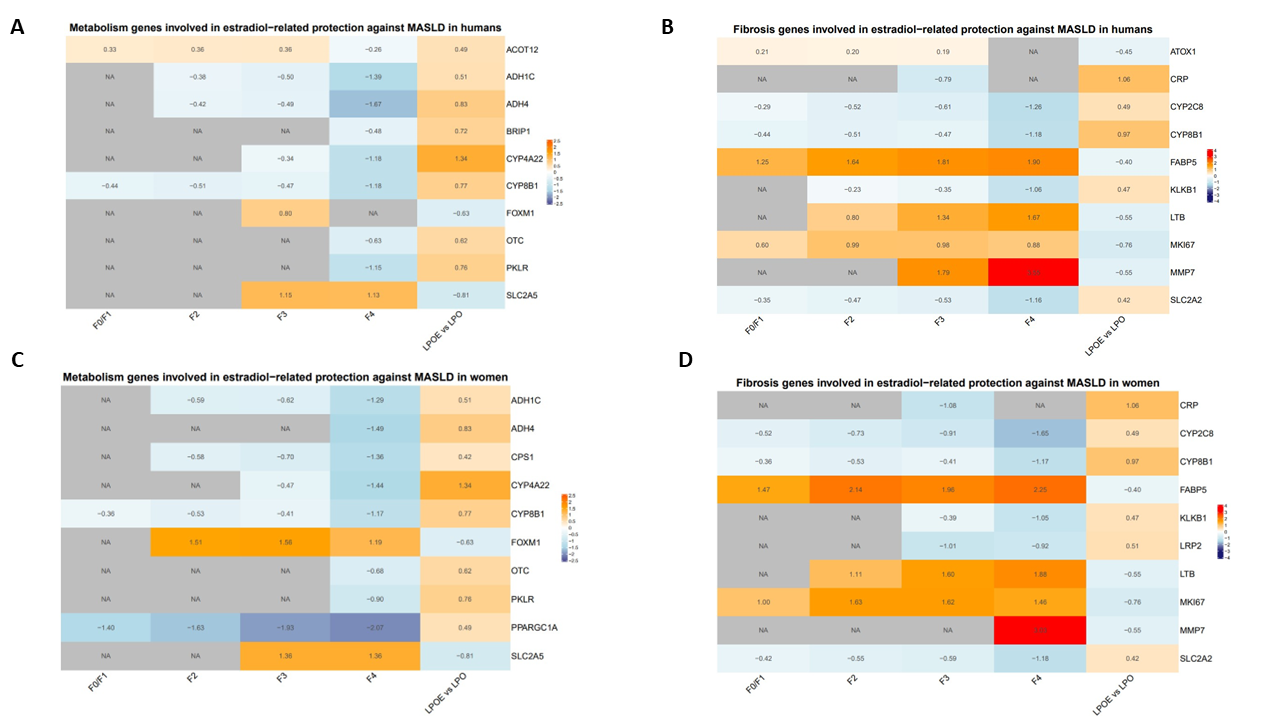

Supplement: Supplementary file 5 — Additional file 5 [file 13287_2025_4238_MOESM5_ESM.tif]

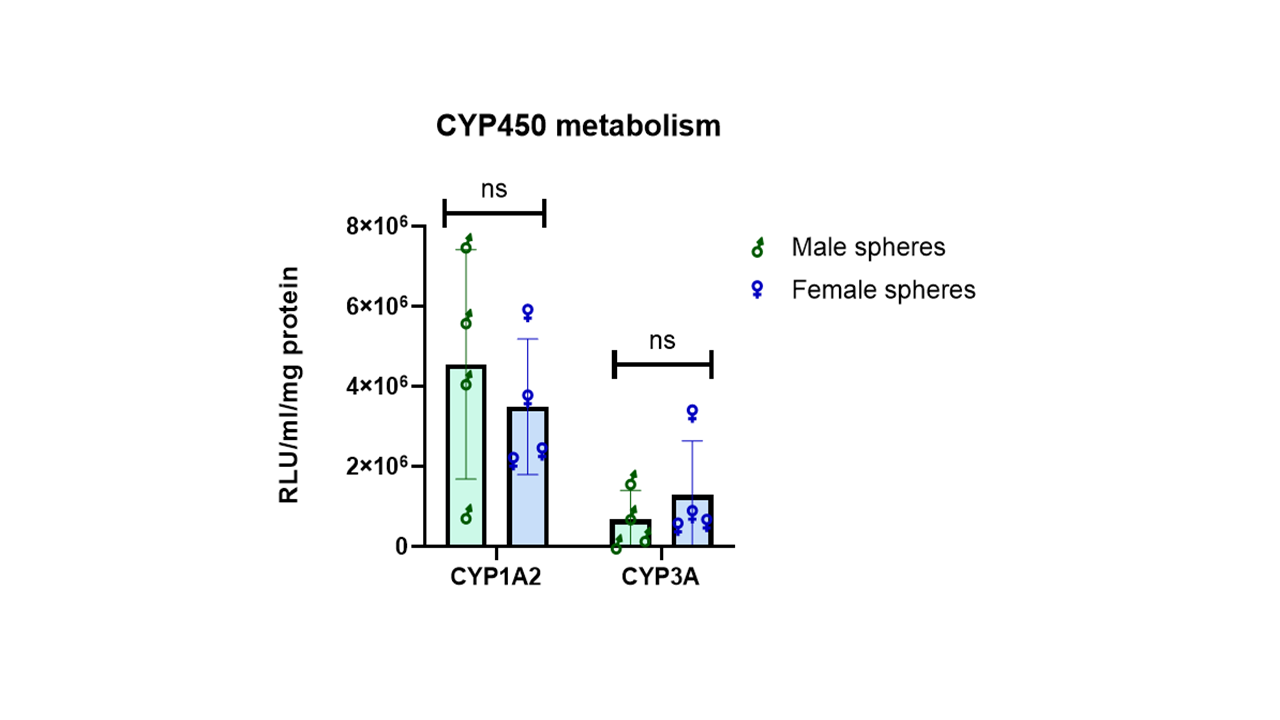

Supplement: Supplementary file 6 — Additional file 6 [file 13287_2025_4238_MOESM6_ESM.tif]

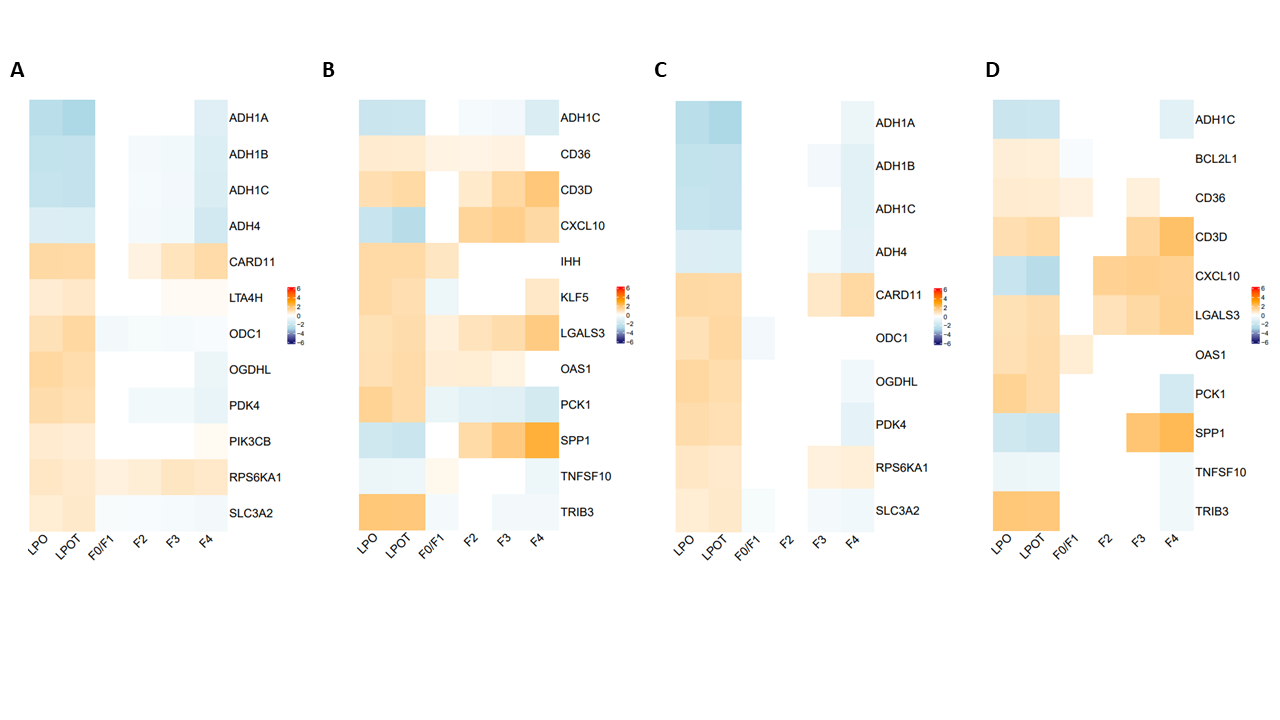

Supplement: Supplementary file 7 — Additional file 7 [file 13287_2025_4238_MOESM7_ESM.tif]

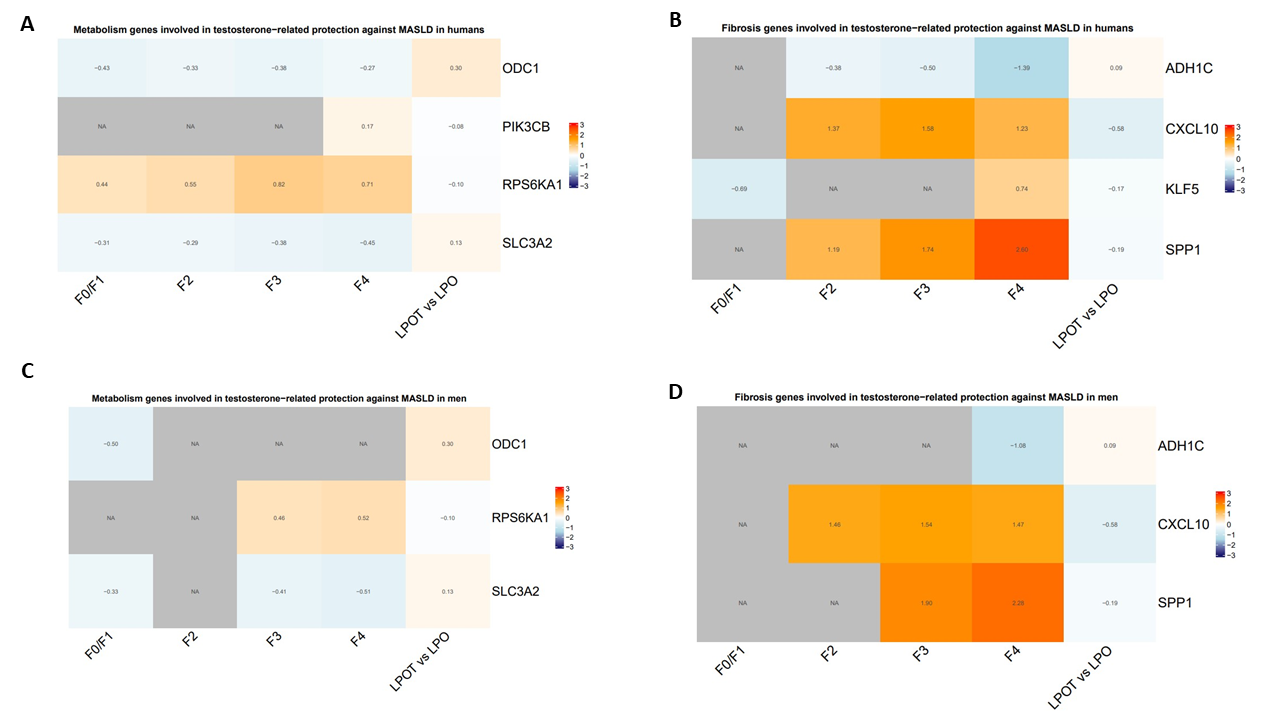

Supplement: Supplementary file 8 — Additional file 8 [file 13287_2025_4238_MOESM8_ESM.tif]

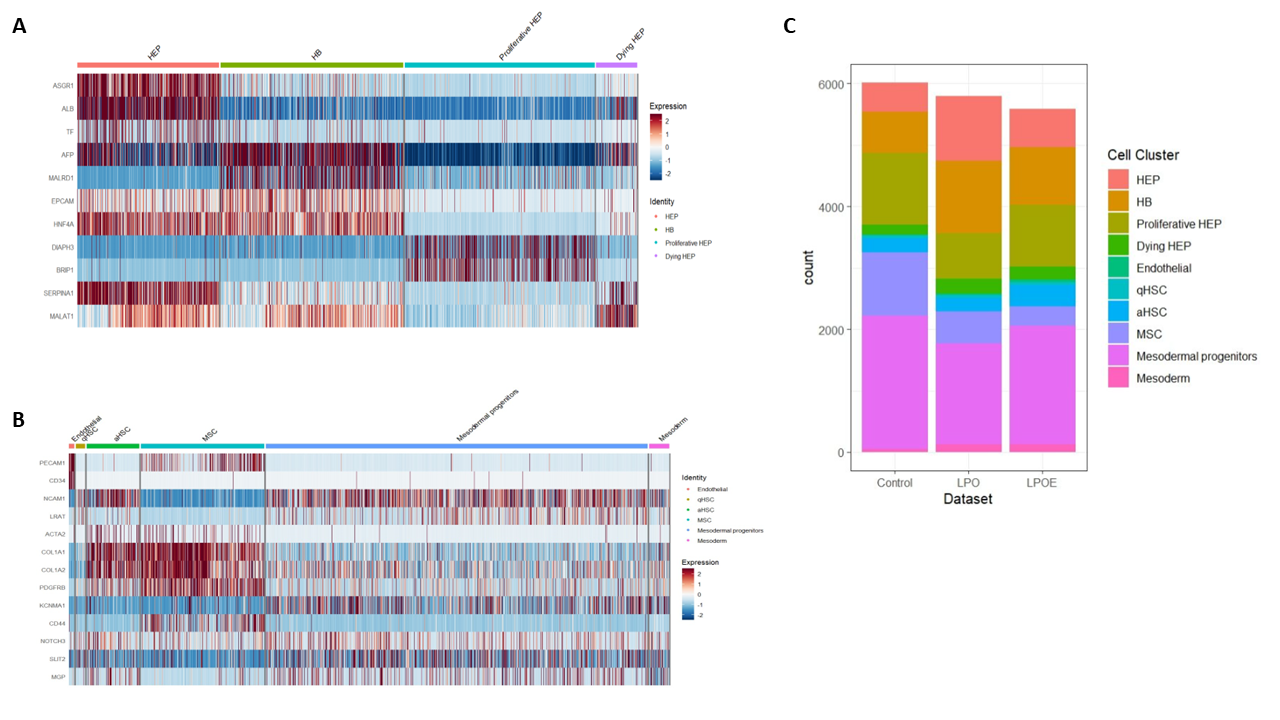

Supplement: Supplementary file 9 — Additional file 9 [file 13287_2025_4238_MOESM9_ESM.tif]

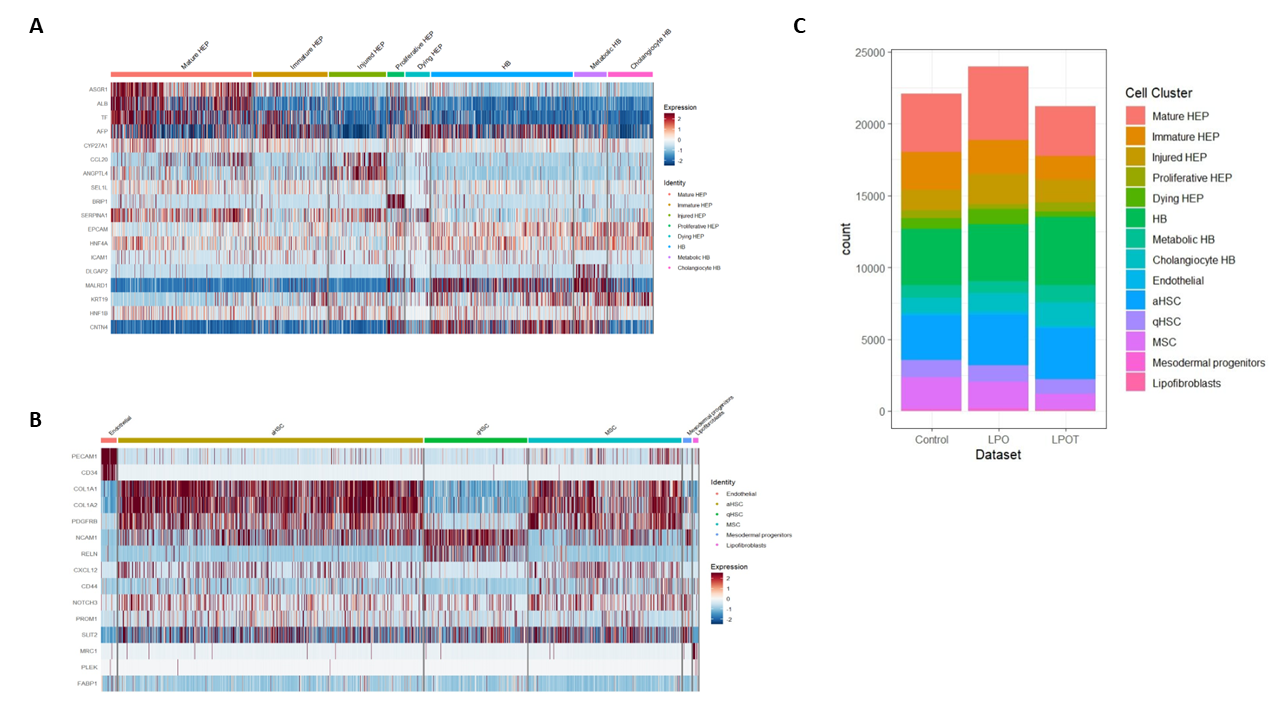

Supplement: Supplementary file 10 — Additional file 10 [file 13287_2025_4238_MOESM10_ESM.tif]
