# Supplementary material for: Hormone correction of dysfunctional metabolic gene expression in stem cell-derived liver tissue
Source: Stem Cell Res Ther. 2025 Mar 11;16:130. doi: 10.1186/s13287-025-04238-0 (PMC11899078; doi:10.1186/s13287-025-04238-0)
Supplement: Supplementary file 41 — Additional file 41 [file 13287_2025_4238_MOESM41_ESM.docx]

**SUPPLEMENTARY FIGURE LEGENDS**

**Supplementary Figure 1.** Characterisation of cells in 2D. (A) Pluripotent stem cell characterisation. PSC cell lines were cultured on Laminin 521 (Biolamina) coated plates in antibiotic free mTESR-Plus^TM^ (STEMCELL Technologies) medium in a humidified 37°C/5% CO_2_ cell culture incubator as previously described^11^. Cells were maintained by continuous passaging using Gentle Cell Dissociation Reagent (STEMCELL Technologies) and replated in small colonies at a dilution of 1:6-1:12. The cells were regularly tested for mycoplasma infection and were reported to be negative. Single-cell populations were resuspended in FACS (Fluoresecent Activated Cell Sorting)-PBS (PBS containing 0.1% BSA and 0.1% sodium azide). The cells were counted and diluted to a density of 1 × 10^6^ viable cells/mL. A total of 2 × 10^5^ cells were incubated for 30 min at 4°C with conjugated antibodies to SSEA-4 and TRA-1-60 (eBioscience). Cells were washed twice in FACS-PBS. Cells incubated with isotype controls were included as control and dead cells and debris were excluded from the analysis based on scatter characteristics. Data for at least 10 000 live events were acquired for each sample using a BD LSR Fortessa cytometer and were analysed using FlowJo version V10 software (FlowJo LLC). Human-specific pluripotent stem cell (PSC) marker SSEA-4 and TRA-1-60 expressions were analysed on PSCs grown in culture for 1 month, with the numbers above the gate indicating the percentage of the cells expressing the markers. Data are presented as percentage positive staining. (B) Immunofluorescent images of PSC marker Oct4 and Nanog double stain, percentages indicating the percentage of cells double expressing these markers (mean ± SD). 2D cell cultures were fixed in 4% paraformaldehyde at 4°C for 30 min. Following this, cells were washed three times 1× PBS at room temperature. Cell monolayers were blocked with PBS-0.1% Tween containing 10% BSA for 1 h, and the monolayers were incubated with primary antibodies diluted in PBS-0.1% Tween/1% BSA at 4 °C overnight. The following day, the primary antibody was removed, and the fixed monolayers were washed three times with 1× PBS-0.1% Tween/1% BSA. Following this, the cells were incubated with the appropriate secondary antibody diluted in PBS/0.1% Tween/1% BSA for 1 h at room temperature and washed three times with 1× PBS. Cultures were counterstained with NucBlue Hoechst 33342 (Sigma-Aldrich) diluted in PBS-0.1% Tween/1% BSA. The cells were imaged with an Opera Phoenix microscope with the 20 X water objective. The images were analysed using SignalsImageArtist (Perkin Elmer). For the quantification of the markers, 9 different fields-of-view (FOV) were selected from each well, containing at least 2000 nuclei per well (n = 6). Represented scale bar is at 50 µm. (C) Quantified data from immunostaining images of d0 PSCs stained with primary control IgG antibodies (3 wells x 9 FOV) and Nanog/Oct4 markers (6 wells x 9 FOV). The graph shows the percentage of all cells expressing the markers, showing the specificity compared to the IgG controls (mean ± SD). (D) PSC differentiation into the three liver cell types used for the assembly of the liver spheres. Hepatic progenitors (HB) are shown at day 0 and day 9 of differentiation (upper panel), hepatic stellate cells (HSC) are shown at day 0 and day 12 of differentiation (middle panel), and endothelial-like cells (EC) are shown at day 0 and day 5 of differentiation (lower panel). The protocol for differentiation to every liver cell type was previously described in ^10,11^. Each image on the right side shows the final day before the harvesting of the monolayer for the liver cell suspension. Phase contrast images were taken at x10 objective with a scale bar of 200 µm. The images were taken using a Phase Contrast via Nikon Eclipse TE300 with the Carl Zeiss lens and were saved using AxioVision Rel. 4.8 software. (E) Immunostaining images of the hepatic progenitors at day 9 of differentiation. 2D cell cultures were fixed with 100% ice-cold methanol at −20 °C for 10 min. Following this, cells were washed three times 1× PBS at room temperature. Cell monolayers were blocked with PBS-0.1% Tween containing 10% BSA for 1 h, and the monolayers were incubated with primary antibodies diluted in PBS-0.1% Tween/1% BSA at 4 °C overnight. The following day, the primary antibody was removed, and the fixed monolayers were washed three times with 1× PBS-0.1% Tween/1% BSA. Following this, the cells were incubated with the appropriate secondary antibody diluted in PBS/0.1% Tween/1% BSA for 1 h at room temperature and washed three times with 1× PBS. Cultures were counterstained with NucBlue Hoechst 33342 (Sigma-Aldrich) diluted in PBS-0.1% Tween/1% BSA. The cells were imaged with an Opera Phoenix microscope with the 20 X water objective. The images were analysed using SignalsImageArtist (Perkin Elmer). Images of hepatocyte nuclear transcription factor 4 alpha (HNF4A), alpha-fetoprotein (AFP) and albumin were quantified using 9 different FOV, containing at least 5000 nuclei per well (n ≥ 4). The percentage of cells expressing those markers (mean ± SD), indicating the early hepatocyte fate with HNF4A and AFP expressions, but no albumin expression. (F) Quantified data from immunostaining images of day 9 hepatic progenitors stained with primary control IgG antibodies (3 wells x 9 FOV) and HNF4A, albumin (ALB) and AFP. The graph shows the percentage of all cells expressing the markers of interest, showing the specificity compared to the IgG controls (mean ± SD). Represented scale bar is at 50 µm. (G) EC characterisation. Single-cell populations were resuspended in FACS (Fluoresecent Activated Cell Sorting)-PBS (PBS containing 0.1% BSA and 0.1% sodium azide). The cells were counted and diluted to a density of 1 × 10^6^ viable cells/mL. A total of 2 × 10^5^ cells were incubated for 30 min at 4°C with conjugated antibodies to CD144 (BD Biosciences, 1:40 dilution). Cells were washed twice in FACS-PBS. Cells incubated with isotype controls were included as control and dead cells and debris were excluded from the analysis based on scatter characteristics. Data for at least 10 000 live events were acquired for each sample using a BD LSR Fortessa cytometer and were analysed using FCS Express 7 Research edition (Windows 64). Following MACS purification of ECs at day 5, the selected population for the spheroids expressed CD144+ (endothelial cell-specific marker). (H) HSC characterisation. Single-cell populations were resuspended in FACS (Fluoresecent Activated Cell Sorting)-PBS (PBS containing 0.1% BSA and 0.1% sodium azide). The cells were counted and diluted to a density of 1 × 10^6^ viable cells/mL. A total of 2 × 10^5^ cells were incubated for 30 min at 4°C with conjugated antibodies to CD140b (BD Biosciences, 1:40 dilution). Cells were washed twice in FACS-PBS. Cells incubated with isotype controls were included as control and dead cells and debris were excluded from the analysis based on scatter characteristics. Data for at least 10 000 live events were acquired for each sample using a BD LSR Fortessa cytometer and were analysed using FCS Express 7 Research edition (Windows 64). Day 12 HSC were analysed using flow cytometry and the expression of stellate marker PDGFRβ was investigated. The percentage above the gates indicates the percentage of cells expressing the marker.

**Supplementary Figure 2.** Sphere aggregation and maintenance. Following the differentiation of hepatic progenitors, endothelial and hepatic stellate cells, the PSC-derived liver cell types were incubated with 1 mL/10 cm^2^ of TrypLE express (Gibco) for 3–20 min at 37°C/5% CO_2_, dependent on the cell type as described previously^10,11^. The dissociation reaction was stopped by adding the same volume of liver sphere medium [50% Williams E medium (Gibco), supplemented with 1% Glutamax, 5% serum replacement (ThermoFisher) and 1% penicillin-streptomycin; 50% Human Endothelial SFM (Gibco), supplemented with 1% penicillin-streptomycin], supplemented with 10 µM Y-27632 (Merck). Single-cell hepatic progenitor and stellate cell suspensions were filtered through 100 μm, then 30 μm strainers to avoid the clumping of the cells. After filtering, the cells were centrifugated at 200 rcf for 5 min. The hepatic progenitor cell pellet were resuspended in liver sphere medium, supplemented with 10 µM Y-27632 (Merck), 10 ng/mL EGF (PeproTech), 10 ng/mL FGF (Peptrotech), 10 ng/mL HGF (Peprotech), 20 ng/mL OSM (Peprotech) and 50 ng/mL VEGF (PeproTech) at a density of 7,68 × 10^6^ live cells/mL for 256-well agarose microplate and 10.95 x 10^6^ cells/mL for 73-microwell SunBioscience hydrogel plate. The hepatic stellate cell pellet was resuspended in the liver sphere medium containing the same growth factors and supplements as described above for the hepatic progenitors at a density of 7.68 × 10^5^ live cells/mL for 256-well agarose microplate and 2.19 x 10^6^ cells/mL for 73-microwell SunBioscience hydrogel plate. After dissociation, the endothelial cells were passed through magnetic-associated cell sorting (MACS) purification for CD144 marker (Myltenyi Biotec) and were resuspended in supplemented liver sphere medium as described for hepatic progenitors and hepatic stellate cells at the density of 5.12 × 10^6^ live cells/mL 256-well agarose microplate and 6.57 x 10^6^ cells/mL for 73-microwell SunBioscience hydrogel plate. The solution of different cell types was prepared at the ratio of 10:3:1 (hepatic progenitor:endothelial cell:hepatic stellate cell), by adding 190 μL of cell suspension to the 256-microwell agarose microplate or 40 μL of cell suspension to the primed 73-microwell SunBioscience hydrogel microplate. The plates were placed in the 37°C/5% CO_2_ cell culture incubator for 2 h to allow the self-aggregation. After the aggregation, 1.5 mL of growth factor supplemented liver medium was added to the 256-microwell agarose microplate containing wells, and 200 μL of growth factor supplemented liver medium was added to the 73-microwell hydrogel plates. The media supplemented with the growth factors was replenished every 48 h. (A) Manual method. Each 12-well plate well has an agarose micromold, where pluripotent stem cell-derived differentiated liver cell suspension is added on top of 256 microwells. Cells sediment by gravity and self-aggregate into liver spheres. Phase contrast image of the established spheres in agarose microwells was taken at x10 magnification, with a scale bar of 200 µm. (B) Semi-automated production of 3D liver spheres. After the cell suspension is prepared, the plates are primed and cells are distributed using multi-drop (left) and Viaflo robot (right) onto the 96 Gri3D plate hydrogels, containing 73 microwells per well. Brightfield image of a hydrogel well was taken at x20 Opera Phoenix objective, creating a Global Image of a full view (scale bar 500 µm). (C) Hepato-endothelial-stellate liver spheres from 1 to 4 weeks in culture. Phase contrast images at x10 magnification (200 µm scale bar) are shown. No cell dissociation from the spheres was observed.

**Supplementary Figure 3.** Medium optimisation and phenotype stabilisation. (A) Liver sphere morphology in different maintenance media. Maintenance medium 1 consisted of Williams E medium (Gibco), supplemented with 1% Glutamax, 5% serum replacement (ThermoFisher) and 1% penicillin-streptomycin; and 10 ng/mL EGF (PeproTech), 10 ng/mL FGF (Peptrotech), 10 ng/mL HGF (Peprotech), 20 ng/mL OSM (Peprotech) and 50 ng/mL VEGF (PeproTech). Whilst Maintenance medium 2 was previously described liver sphere medium [50% Williams E medium (Gibco), supplemented with 1% Glutamax, 5% serum replacement (ThermoFisher) and 1% penicillin-streptomycin; 50% Human Endothelial SFM (Gibco), supplemented with 1% penicillin-streptomycin], 10 ng/mL EGF (PeproTech), 10 ng/mL FGF (Peptrotech), 10 ng/mL HGF (Peprotech), 20 ng/mL OSM (Peprotech) and 50 ng/mL VEGF (PeproTech). Phase contrast images taken at x10 objective show differences in morphology when grown in maintenance medium 1 (upper panel) and maintenance medium 2 (bottom panel) at 8 weeks in culture (scale bar 200 µm). Hepato- (H), hepato-endothelial (HE) and hepato-endothelial-stellate liver spheres were compared in both media at 8 weeks. Maintenance medium 2 showed larger and more developed HE and HES spheres than maintenance medium 1. (B) Liver sphere metabolic activity in different maintenance media. Hepato- (H), hepato-endothelial (HE) and hepato-endothelial-stellate (HES) liver sphere cytochrome P450 1A2 (CYP1A2) and 3A (CYP3A) activities (RLU/mL) at week 8 were compared between maintenance medium 1 and maintenance medium 2 (mean ± SD, n = 4). Within maintenance medium 2, HE and HES liver spheres remain metabolically active even after 8 weeks. There was a significant difference in HE and HES CYP1A2 (blue) and CYP3A (purple) activity compared to the H activity within maintenance medium 2, suggesting that the addition of other cell types (endothelial and/or stellate cells) is required for sphere stability and metabolic function long term. Data was normalised on the total protein content per sample (mg protein) and different liver tissue compositions were compared within the media using two-way analysis of variance (ANOVA) with Tukey’s multiple comparisons test to assess the P-value. (C) Liver sphere viability in long-term culture. Hepato-endothelial-stellate liver sphere viability (RLU/mL) was measured at weeks 4 and 8 (mean ± SD, n = 4) using RealTime-Glo MT Cell Viability Assay (Promega) following the manufacturer's instructions, at 24 h post exposure as described previously^10^. Results were normalised by protein content (mg) measured using BCA assay (ThermoFisher). Liver spheres grown in maintenance medium 2 showed to be as viable at week 8 as they were at week 4. P-values are indicated as follows: * (P < 0.05), ** (P ≤ 0.01) and **** (P ≤ 0.0001).

**Supplementary Figure 4.** Modelling MASLD *in vitro* in females. Female liver spheres were incubated with 17β-estradiol (E2, 10^-8^ M) for 7 days (week 3) before incubation with LPO. RNAs for control, E2, LPO and LPOE treatments (n=3; 1 x n = 768 spheres) were extracted using QIAzol Lysis Reagent (Qiagen) and RNeasy MiniPrep (Qiagen) as per manufacturer’s instructions. The RNA quality was assessed using Bioanalyzer (Agilent). The gene expression was assessed using NanoString nCounter technology via fibrosis (770 genes, 51 pathways) and Metabolism (768 genes) panels (NanoString). All the treatments were compared with the vehicle spheres (control). Only the genes with the differential expressions from LPO and LPO with the hormone were compared with the differential gene expression from F0/F1, F2, F3 and F4 stage fibrosis patients from SteatoSITE bulk-RNA sequencing, since hormone-only control was not significantly different from normal control. Differentially expressed genes in LPO and LPOE were selected by an absolute log_2_FC ≥ 0.5 (|log_2_FC| ≥ 0.5) and an adjusted P-value < 0.05 according to the Benjamini-Yekutieli procedure. Then, the selected genes were compared with the whole human bulk-RNA sequencing data available in SteatoSITE (n=650), covering the complete MASLD spectrum (against all fibrosis stages (F0/F1 to F4)^12^, compared with normal liver controls, where one or more of the stages had a significant gene expression change for the gene (adjusted P value < 0.05 according to Benjamini-Hochberg procedure). The heatmaps where produced using R v.4.3.0. The Spearman correlation analysis was performed between the whole SteatoSITE and gender-specific SteatoSITE against the LPO and LPOE, and the significant (p. value < 0.05) correlation coefficients (ρ) were selected. (A) Metabolism-associated gene expression comparison between female spheres and all stages of human liver fibrosis shown in a heatmap based on their log_2_FC values. (B) Fibrosis-associated gene expression comparison between female spheres and all stages of human liver fibrosis shown in a heatmap based on their log_2_FC values. (C) Metabolism-associated gene expression comparison between female spheres and all stages of women fibrosis in MASLD shown in a heatmap based on their log_2_FC values. Experiments and comparisons were performed as mentioned above. However, instead of the full patient data, the female sphere results from LPO and LPOE were compared only with SteatoSITE RNA-sequencing data from women cohort. (D) Fibrosis-associated gene expression comparison between female spheres and all stages of women fibrosis in MASLD shown in a heatmap based on their log_2_FC values. Experiments and comparisons were performed as mentioned. However, instead of the full patient data, the female sphere results from LPO and were compared only with SteatoSITE RNA-sequencing data from women cohort. Upregulated genes are visualised on a colour scale from white to red (log_2_FC values from 0 to 6), whilst downregulated genes are visualised on a colour scale from white to blue (log_2_FC values from 0 to -6) in all panels.

**Supplementary Figure 5**. Impact of E2 on expression of genes implicated in development of MASLD in liver spheres derived from female PSCs. Female liver spheres were incubated with 17β-estradiol (E2, 10^-8^ M) for 7 days (week 3) before incubation with LPO. Expression of genes associated with metabolism and fibrosis for all treatments (n=3; 1 x n = 768 spheres) was assessed using NanoString nCounter technology as described in the Supplementary Figure 4 legend. All the treatments were compared with the vehicle spheres (control). Differentially expressed genes in LPO and LPOE were selected by an absolute log_2_FC ≥ 0.5 (|log_2_FC| ≥ 0.5) and an adjusted P-value < 0.05 according to the Benjamini-Yekutieli procedure. Then, the selected genes were compared with the whole human bulk-RNA sequencing data available in SteatoSITE (n=650), covering the complete MASLD spectrum (against all fibrosis stages (F0/F1 to F4), compared with normal liver controls, where one or more of the stages had a significant gene expression change for the gene (adjusted P value < 0.05 according to Benjamini-Hochberg procedure). Following this, the biggest difference between significant LPO and LPOE genes was assessed (|log_2_FC(LPOE) - log_2_FC(LPO)|), when compared to control spheres, and those with improved expression pattern after hormone treatment were identified after comparison with the expression pattern in late fibrosis (F3-F4) stages (log_2_FC) were selected. The log_2_FC values were plotted on a heatmap as well as log_2_FC value of difference between LPOE and LPO treatments compared to controls. (A) Impact of E2 pre-treatment on expression of metabolism-associated genes compared to whole human bulk-RNA sequencing data as described above, shown in a heatmap with log_2_FC values. Upregulation is visualised on a colour scale from white to red (log_2_FC values from 0 to 2.5), whilst downregulation is visualised on a colour scale from white to blue (log_2_FC values from 0 to -2.5). (B) Impact of E2 pre-treatment on fibrosis-associated gene compared to whole human bulk-RNA sequencing data as described above, shown in a heatmap with log_2_FC values. Upregulation is visualised on a colour scale from white to red (log_2_FC values from 0 to 4), whilst downregulation is visualised on a colour scale from white to blue (log_2_FC values from 0 to -4). (C) Metabolism-associated gene signatures involved in E2-related protection against MASLD development in women shown in a heatmap with log_2_FC values. Experiments and comparisons were performed as mentioned above. However, instead of the whole SteatoSITE patient data, the female sphere results were compared only with SteatoSITE RNA-sequencing data from women cohort. Upregulated genes are visualised on a colour scale from white to red (log_2_FC values from 0 to 2.5), whilst downregulated genes are visualised on a colour scale from white to blue (log_2_FC values from 0 to -2.5). (D) Fibrosis-associated gene expression comparison between female spheres and all stages of women fibrosis in MASLD shown in a heatmap with log_2_FC values. Experiments and comparisons were performed as mentioned above. However, instead of the full patient data, the female sphere results were compared only with SteatoSITE RNA-sequencing data from women cohort. Upregulated genes are visualised on a colour scale from white to red (log_2_FC values from 0 to 4), whilst downregulated genes are visualised on a colour scale from white to blue (log_2_FC values from 0 to -4).

**Supplementary Figure 6.** Metabolic equivalence in liver spheres. Week 3-4 liver spheres derived from either male (green) PSCs or female (blue) PSCs were assessed by measuring their cytochrome P450 (CYP450) activities (mean ± SD, n = 4) as described previously in Figure 1 legend and ^10,11^. No significant differences between male and female liver spheres CYP1A2 and CYP3A metabolism. Differences in CYP1A2 and CYP3A activity between male and female spheres were investigated by two-way analysis of variance (ANOVA) with Šídák's multiple comparisons test for the P-value evaluation. P-values are indicated as follows: ns (P > 0.05), determined as non-significant.

**Supplementary Figure 7.** Modelling MASLD *in vitro* in males. Male liver spheres were incubated with testosterone (T, 10^-8^ M) for 7 days (week 3) before incubation with LPO. RNAs for control, T, LPO and LPOT treatments (n=3; 1 x n = 768 spheres) were extracted using QIAzol Lysis Reagent (Qiagen) and RNeasy MiniPrep (Qiagen) as per manufacturer’s instructions. The RNA quality was assessed using Bioanalyzer (Agilent). The gene expression was assessed using NanoString nCounter technology via fibrosis (770 genes, 51 pathways) and Metabolism (768 genes) panels (NanoString). All the treatments were compared with the vehicle spheres (control). Only the genes with the differential expressions from LPO and LPOT were compared with the differential gene expression from F0/F1, F2, F3 and F4 stage fibrosis patients from SteatoSITE bulk-RNA sequencing, since hormone-only control was not significantly different from normal control. Differentially expressed genes in LPO and LPOT were selected by an absolute log_2_FC ≥ 0.5 (|log_2_FC| ≥ 0.5) and an adjusted P-value < 0.05 according to the Benjamini-Yekutieli procedure. Then, the selected genes were compared with the whole human bulk-RNA sequencing data available in SteatoSITE (n=650), covering the complete MASLD spectrum (against all fibrosis stages (F0/F1 to F4)^12^, compared with normal liver controls, where one or more of the stages had a significant gene expression change for the gene (adjusted P value < 0.05 according to Benjamini-Hochberg procedure). The heatmaps where produced using R v.4.3.0. (A) Metabolism-associated gene expression comparison between male spheres and all stages of human liver fibrosis shown in a heatmap based on their log_2_FC values. (B) Fibrosis-associated gene expression comparison between male spheres and all stages of human liver fibrosis shown in a heatmap based on their log_2_FC values. (C) Metabolism-associated gene expression comparison between male spheres and all stages of MASLD-associated fibrosis in men. Experiments and comparisons were performed as mentioned above. However, instead of the full patient data, the male sphere results from LPO and LPOT were compared only with SteatoSITE RNA-sequencing data from men cohort. (D) Fibrosis-associated gene expression comparison between male spheres and all stages of MASLD-associated fibrosis in men shown in a heatmap based on their log_2_FC values. Experiments and comparisons were performed as mentioned above. However, instead of the full patient data, the male sphere results from LPO and LPOT were compared only with SteatoSITE RNA-sequencing data from men cohort. Upregulated genes are visualised on a colour scale from white to red (log_2_FC values from 0 to 6), whilst downregulated genes are visualised on a colour scale from white to blue (log_2_FC values from 0 to -6) in all panels.

**Supplementary Figure 8**. Impact of T on expression of genes implicated in development MASLD in liver spheres derived from male PSCs. Male liver spheres were incubated testosterone (T, 10^-8^ M) for 7 days (week 3) before incubation with LPO. Expression of genes associated with metabolism and fibrosis for all treatments (n=3; 1 x n = 768 spheres) was assessed using NanoString nCounter technology. All the treatments were compared with the vehicle spheres (control). Differentially expressed genes in LPO and LPOT were selected by an absolute log_2_FC ≥ 0.5 (|log_2_FC| ≥ 0.5) and an adjusted P-value < 0.05 according to the Benjamini-Yekutieli procedure. Then, the selected genes were compared with the whole human bulk-RNA sequencing data available in SteatoSITE (n=650), covering the complete MASLD spectrum (against all fibrosis stages (F0/F1 to F4), compared with normal liver controls, where one or more of the stages had a significant gene expression change for the gene (adjusted P value < 0.05 according to Benjamini-Hochberg procedure). Following this, the biggest difference between significant LPO and LPOT genes was assessed (|log_2_FC(LPOT) - log_2_FC(LPO)|), when compared to control spheres, and those with improved expression pattern after hormone treatment were identified after comparison with the expression pattern in late fibrosis (F3-F4) stages (log_2_FC) were selected. The log_2_FC values were plotted on a heatmap as well as log_2_FC value of difference between LPOE and LPO treatments compared to controls. (A) Metabolism-associated gene signatures involved in T-related protection against MASLD development in humans shown in a heatmap with log_2_FC values. (B) Fibrosis-associate gene signatures involved in T-related protection against MASLD development in humans shown in a heatmap with log_2_FC values. (C) Metabolism-associated gene signatures involved in T-related protection against MASLD development in men shown in a heatmap with log_2_FC values. Experiments and comparisons were performed as mentioned above. However, instead of the full patient data, the male sphere results were compared only with SteatoSITE RNA-sequencing data from men cohort. (D) Fibrosis-associated gene signatures involved in T-related protection against MASLD development in men shown in a heatmap with log_2_FC values. Experiments and comparisons were performed as mentioned above. However, instead of the full patient data, the male sphere results were compared only with SteatoSITE RNA-sequencing data from men cohort. Upregulated genes are visualised on a colour scale from white to red (log_2_FC values from 0 to 3), whilst downregulated genes are visualised on a colour scale from white to blue (log_2_FC values from 0 to -3) in all panels.

**Supplementary Figure 9.** Female liver spheres single nuclei RNA sequencing cluster identification. (A) Heatmap showing the expression for known markers for parenchymal clusters. ASGR1, ALB and TF were upregulated in hepatocytes (HEP); AFP, MALRD1, EPCAM and HNF4A were upregulated in hepatic progenitors (HB); DIAPH3 and BRIP1 were upregulated in proliferative hepatocytes (Proliferative HEP); and SERPINA1 was upregulated in dying hepatocytes (Dying HEP). (B) Heatmap showing the expression for known markers for non-parenchymal clusters. PECAM1 and CD34 were upregulated in homogenic endothelium (Endothelial); NCAM1 and LRAT were upregulated in quiescent stellate cells (qHSC); ACTA2, COL1A1 and COL1A2 were upregulated in activated stellate cells (aHSC); CD44 and NOTCH3 were upregulated in mesenchymal stromal cells (MSC); NOTCH3, SLIT2 and MGP were upregulated in mesodermal progenitors; and other mesoderm cells expressed most of the above markers except from PECAM1, CD34, ACTA2 and CD44. (C) Bar plot showing the cell count for each dataset within separate clusters. Most of the cells were from Mesodermal progenitor cluster, followed by hepatic progenitors (HB), hepatocytes (HEP) and mesenchymal stromal cell (MSC) clusters in each dataset. However, there were differences in cell numbers in specific clusters between different treatment conditions.

**Supplementary figure 10.** Male liver sphere single-nuclei RNA sequencing cluster identification. (A) Heatmap showing the expression for known markers for parenchymal clusters. ASGR1, ALB and TF were upregulated in mature hepatocytes (Mature HEP); AFP and CYP27A1 were upregulated in immature hepatocytes (Immature HEP); CCL20 and ANGTPL4 were upregulated in injured hepatocytes (Injured HEP); SEL1L1 and BRIP1 were upregulated in proliferative hepatocytes (Proliferative HEP); and SERPINA1 was upregulated in dying hepatocytes (Dying HEP). EPCAM, HNF4, AFP and ICAM1 were upregulated in hepatic progenitors (HB); DLGAP2 and MALRD1 were upregulated in hepatic progenitors associated with metabolism in liver (Metabolic HB); KRT19, HNF4A, HNF1B but not CNTN were upregulated in cholangiocyte-like hepatic progenitors (Cholangiocyte HB). (B) Heatmap showing the expression for known markers for non-parenchymal clusters. PECAM1 and CD34 were upregulated in homogenic endothelium (Endothelial); COL1A1, COL1A2 and PDGFRB were upregulated in activated stellate cells (aHSC); NCAM1 and RELN were upregulated in quiescent stellate cells (qHSC); CXCL12 and CD44 were upregulated in mesenchymal stromal cells (MSC); CD44, NOTCH3, PROM1 and SLIT2 were upregulated in mesodermal progenitors; and CD44, MRC1, PLEK and FABP5 were upregulated in lipofibroblasts. (C) Bar plot showing the cell count for each dataset within separate cell clusters. The majority of the cells were mature hepatocytes and hepatic progenitors in each dataset. However, there were differences in cell numbers in specific clusters between different treatment conditions.

**Supplementary Table 1.** Metabolism-associated gene expression comparison between female spheres and all stages of human liver fibrosis. Female liver spheres were incubated with 17β-estradiol (E2, 10-8 M) for 7 days (week 3) before incubation with LPO. Expression of genes associated with metabolism for all treatments (n=3; 1 x n = 768 spheres) was assessed using NanoString nCounter technology. All the treatments were compared with the vehicle spheres (control). Differentially expressed genes in LPO and LPOE were selected by an absolute log2FC ≥ 0.5 (|log2FC| ≥ 0.5) and an adjusted P-value < 0.05 according to the Benjamini-Yekutieli procedure. Then, the selected genes were compared with the whole human bulk-RNA sequencing data available in SteatoSITE (n=650), covering the complete MASLD spectrum (against all fibrosis stages (F0/F1 to F4), compared with normal liver controls, where one or more of the stages had a significant gene expression change for the gene (adjusted P value < 0.05 according to Benjamini-Hochberg procedure).

**Supplementary Table 2.** Fibrosis-associated gene expression comparison between female spheres and all stages of human liver fibrosis. Female liver spheres were incubated with 17β-estradiol (E2, 10-8 M) for 7 days (week 3) before incubation with LPO. Expression of genes associated with fibrosis for all treatments (n=3; 1 x n = 768 spheres) was assessed using NanoString nCounter technology. All the treatments were compared with the vehicle spheres (control). Differentially expressed genes in LPO and LPOE were selected by an absolute log2FC ≥ 0.5 (|log2FC| ≥ 0.5) and an adjusted P-value < 0.05 according to the Benjamini-Yekutieli procedure. Then, the selected genes were compared with the whole human bulk-RNA sequencing data available in SteatoSITE (n=650), covering the complete MASLD spectrum (against all fibrosis stages (F0/F1 to F4), compared with normal liver controls, where one or more of the stages had a significant gene expression change for the gene (adjusted P value < 0.05 according to Benjamini-Hochberg procedure).

**Supplementary Table 3.** Metabolism-associated gene expression comparison between female spheres and all stages of MASLD-associated fibrosis in women. Female liver spheres were incubated with 17β-estradiol (E2, 10-8 M) for 7 days (week 3) before incubation with LPO. Expression of genes associated with metabolism for all treatments (n=3; 1 x n = 768 spheres) was assessed using NanoString nCounter technology. All the treatments were compared with the vehicle spheres (control). Differentially expressed genes in LPO and LPOE were selected by an absolute log2FC ≥ 0.5 (|log2FC| ≥ 0.5) and an adjusted P-value < 0.05 according to the Benjamini-Yekutieli procedure. Then, the selected genes were compared with the bulk-RNA sequencing data from women cohort available in SteatoSITE (n=650), covering the complete MASLD spectrum (against all fibrosis stages (F0/F1 to F4), compared with normal liver controls, where one or more of the stages had a significant gene expression change for the gene (adjusted P value < 0.05 according to Benjamini-Hochberg procedure).

**Supplementary Table 4.** Fibrosis-associated gene expression comparison between female spheres and all stages of MASLD-associated fibrosis in women. Female liver spheres were incubated with 17β-estradiol (E2, 10-8 M) for 7 days (week 3) before incubation with LPO. Expression of genes associated with fibrosis for all treatments (n=3; 1 x n = 768 spheres) was assessed using NanoString nCounter technology. All the treatments were compared with the vehicle spheres (control). Differentially expressed genes in LPO and LPOE were selected by an absolute log2FC ≥ 0.5 (|log2FC| ≥ 0.5) and an adjusted P-value < 0.05 according to the Benjamini-Yekutieli procedure. Then, the selected genes were compared with the bulk-RNA sequencing data from women cohort available in SteatoSITE (n=650), covering the complete MASLD spectrum (against all fibrosis stages (F0/F1 to F4), compared with normal liver controls, where one or more of the stages had a significant gene expression change for the gene (adjusted P value < 0.05 according to Benjamini-Hochberg procedure).

**Supplementary Table 5.** Metabolism-associated gene expression comparison between male spheres and all stages of human liver fibrosis. Male liver spheres were incubated with testosterone (T, 10-8 M) for 7 days (week 3) before incubation with LPO. Expression of genes associated with metabolism for all treatments (n=3; 1 x n = 768 spheres) was assessed using NanoString nCounter technology. All the treatments were compared with the vehicle spheres (control). Differentially expressed genes in LPO and LPOT were selected by an absolute log2FC ≥ 0.5 (|log2FC| ≥ 0.5) and an adjusted P-value < 0.05 according to the Benjamini-Yekutieli procedure. Then, the selected genes were compared with the whole human bulk-RNA sequencing data available in SteatoSITE (n=650), covering the complete MASLD spectrum (against all fibrosis stages (F0/F1 to F4), compared with normal liver controls, where one or more of the stages had a significant gene expression change for the gene (adjusted P value < 0.05 according to Benjamini-Hochberg procedure).

**Supplementary Table 6.** Fibrosis-associated gene expression comparison between male spheres and all stages of human liver fibrosis. Male liver spheres were incubated with testosterone (T, 10-8 M) for 7 days (week 3) before incubation with LPO. Expression of genes associated with fibrosis for all treatments (n=3; 1 x n = 768 spheres) was assessed using NanoString nCounter technology. All the treatments were compared with the vehicle spheres (control). Differentially expressed genes in LPO and LPOT were selected by an absolute log2FC ≥ 0.5 (|log2FC| ≥ 0.5) and an adjusted P-value < 0.05 according to the Benjamini-Yekutieli procedure. Then, the selected genes were compared with the whole human bulk-RNA sequencing data available in SteatoSITE (n=650), covering the complete MASLD spectrum (against all fibrosis stages (F0/F1 to F4), compared with normal liver controls, where one or more of the stages had a significant gene expression change for the gene (adjusted P value < 0.05 according to Benjamini-Hochberg procedure).

**Supplementary Table 7.** Metabolism-associated gene expression comparison between female spheres and all stages of MASLD-associated fibrosis in men. Male liver spheres were incubated with testosterone (T, 10-8 M) for 7 days (week 3) before incubation with LPO. Expression of genes associated with metabolism for all treatments (n=3; 1 x n = 768 spheres) was assessed using NanoString nCounter technology. All the treatments were compared with the vehicle spheres (control). Differentially expressed genes in LPO and LPOT were selected by an absolute log2FC ≥ 0.5 (|log2FC| ≥ 0.5) and an adjusted P-value < 0.05 according to the Benjamini-Yekutieli procedure. Then, the selected genes were compared with the bulk-RNA sequencing data from men cohort available in SteatoSITE (n=650), covering the complete MASLD spectrum (against all fibrosis stages (F0/F1 to F4), compared with normal liver controls, where one or more of the stages had a significant gene expression change for the gene (adjusted P value < 0.05 according to Benjamini-Hochberg procedure).

**Supplementary Table 8.** Fibrosis-associated gene expression comparison between male spheres and all stages of MASLD-associated fibrosis in men. Male liver spheres were incubated with testosterone (T, 10-8 M) for 7 days (week 3) before incubation with LPO. Expression of genes associated with metabolism and fibrosis for all treatments (n=3; 1 x n = 768 spheres) was assessed using NanoString nCounter technology. All the treatments were compared with the vehicle spheres (control). Differentially expressed genes in LPO and LPOT were selected by an absolute log2FC ≥ 0.5 (|log2FC| ≥ 0.5) and an adjusted P-value < 0.05 according to the Benjamini-Yekutieli procedure. Then, the selected genes were compared with the bulk-RNA sequencing data from men cohort available in SteatoSITE (n=650), covering the complete MASLD spectrum (against all fibrosis stages (F0/F1 to F4), compared with normal liver controls, where one or more of the stages had a significant gene expression change for the gene (adjusted P value < 0.05 according to Benjamini-Hochberg procedure).

**Supplementary Table 9.** Female single nuclei RNA-sequencing metadata. Sequenced raw data was processed using CellRanger v.7.1.0 and CellRanger count pipeline. Downstream analyses were performed in R (v.4.3.0) using Seurat package (v.4.3.0.) Nuclei with less than 200 and more than 8,000 RNA features, and with more than 20% mitochondrial transcripts were excluded from the downstream analysis. Each gene expression value was log normalised by a total expression in the corresponding nuclei and then multiplied by a scaling factor of 10,000. We then calculated a subset of features exhibiting a high cell-to-cell variability within the dataset, returning 3,000 features per dataset using the linear relationship of log(variance) and log(mean) using polynomial regression (loess), calculating the feature variance on the standardised values after clipping to a maximum. We further applied a linear transformation, scaling, with only variable features being scaled. We inspected and regressed the data based on the cell cycle heterogeneity as well. All the datasets belonging to the H9-derived (female) conditions were merged into a single Seurat object and they were normalised again using SCTransform function. For dimensionality reduction Harmony (v.0.1.1) was used on the top 30 significant principal components. Clustering was performed on 8 different resolutions (0.1 – 0.8) and the relationship between clusters and the resolution was observed using Clustree138 (v.0.5.0). The 0.3 resolution was selected for cluster analysis. The conserved genes between the datasets (control, LPO, LPOE) for females were assessed and top genes were compared to signatures available in the literature. In addition, the cell-specific gene marker expressions from the literature were investigated. The clusters were relabelled as noted in “new_id” column of the metadata.

**Supplementary Table 10.** The most differentially expressed hepatocyte (HEP) genes between LPO (compared to control) and LPOE (compared to control) (|log2FC(LPO)-(log2FC(LPOE)| ≥ 0.5, adjusted P-value < 0.05 according to Bonferroni correction) from female liver spheres.

**Supplementary Table 11.** The most differentially expressed genes from female sphere hepatocyte (HEP) cluster were compared to SteatoSITE bulk RNA-sequencing from women patients. Differential analysis was carried out with ‘limma-voom’ (v.3.28.14) with the protein-coding genes. Statistical significance of genes was determined by an adjusted P-value according to the Benjamini-Hochberg procedure of p < 0.05.

**Supplementary Table 12.** The most differentially expressed hepatic progenitor (HB) genes between LPO (compared to control) and LPOE (compared to control) (|log2FC(LPO)-(log2FC(LPOE)| ≥ 0.5, adjusted P-value < 0.05 according to Bonferroni correction) from female liver spheres.

**Supplementary Table 13.** The most differentially expressed genes from female sphere hepatic progenitor (HB) cluster were compared to SteatoSITE bulk RNA-sequencing from women patients. Differential analysis was carried out with ‘limma-voom’ (v.3.28.14) with the protein-coding genes. Statistical significance of genes was determined by an adjusted P-value according to the Benjamini-Hochberg procedure of p < 0.05.

**Supplementary Table 14.** The most differentially expressed dying hepatocyte (Dying HEP) genes between LPO (compared to control) and LPOE (compared to control) (|log2FC(LPO)-(log2FC(LPOE)| ≥ 0.5, adjusted P-value < 0.05 according to Bonferroni correction) from female liver spheres.

**Supplementary Table 15.** The most differentially expressed genes from female sphere dying hepatocyte (Dying HEP) cluster were compared to SteatoSITE bulk RNA-sequencing from women patients. Differential analysis was carried out with ‘limma-voom’ (v.3.28.14) with the protein-coding genes. Statistical significance of genes was determined by an adjusted P-value according to the Benjamini-Hochberg procedure of p < 0.05.

**Supplementary Table 16.** The most differentially expressed mesodermal progenitor genes between LPO (compared to control) and LPOE (compared to control) (|log2FC(LPO)-(log2FC(LPOE)| ≥ 0.5, adjusted P-value < 0.05 according to Bonferroni correction) from female liver spheres.

**Supplementary Table 17.** The most differentially expressed genes from female sphere mesodermal progenitor cluster were compared to SteatoSITE bulk RNA-sequencing from women patients. Differential analysis was carried out with ‘limma-voom’ (v.3.28.14) with the protein-coding genes. Statistical significance of genes was determined by an adjusted P-value according to the Benjamini-Hochberg procedure of p < 0.05.

**Supplementary Table 18.** The most differentially expressed mesenchymal stromal cell (MSC) genes between LPO (compared to control) and LPOE (compared to control) (|log2FC(LPO)-(log2FC(LPOE)| ≥ 0.5, adjusted P-value < 0.05 according to Bonferroni correction) from female liver spheres.

**Supplementary Table 19.** The most differentially expressed genes from female sphere mesenchymal stromal cell (MSC) cluster were compared to SteatoSITE bulk RNA-sequencing from women patients. Differential analysis was carried out with ‘limma-voom’ (v.3.28.14) with the protein-coding genes. Statistical significance of genes was determined by an adjusted P-value according to the Benjamini-Hochberg procedure of p < 0.05.

**Supplementary Table 20.** Male single nuclei RNA-sequencing metadata. Sequenced raw data was processed using CellRanger v.7.1.0 and CellRanger count pipeline. Downstream analyses were performed in R (v.4.3.0) using Seurat package (v.4.3.0.) Nuclei with less than 200 and more than 8,000 RNA features, and with more than 20% mitochondrial transcripts were excluded from the downstream analysis. Each gene expression value was log normalised by a total expression in the corresponding nuclei and then multiplied by a scaling factor of 10,000. We then calculated a subset of features exhibiting a high cell-to-cell variability within the dataset, returning 3,000 features per dataset using the linear relationship of log(variance) and log(mean) using polynomial regression (loess), calculating the feature variance on the standardised values after clipping to a maximum. We further applied a linear transformation, scaling, with only variable features being scaled. We inspected and regressed the data based on the cell cycle heterogeneity as well. All the datasets belonging to the p106-derived (male) conditions were merged into a single Seurat object and they were normalised again using SCTransform function. For dimensionality reduction Harmony (v.0.1.1) was used on the top 30 significant principal components. Clustering was performed on 8 different resolutions (0.1 – 0.8) and the relationship between clusters and the resolution was observed using Clustree138 (v.0.5.0). The 0.3 resolution was selected for cluster analysis. The conserved genes between the datasets (control, LPO, LPOT) for males were assessed and top genes were compared to signatures available in the literature. In addition, the cell-specific gene marker expressions from the literature were investigated. The clusters were relabelled as noted in “new_id” column of the metadata.

**Supplementary Table 21.** The most differentially expressed hepatocyte (HEP) genes between LPO (compared to control) and LPOT (compared to control) (|log2FC(LPO)-(log2FC(LPOT)| ≥ 0.5, adjusted P-value < 0.05 according to Bonferroni correction) from male liver spheres.

**Supplementary Table 22.** The most differentially expressed genes from male sphere hepatocyte (HEP) cluster were compared to SteatoSITE bulk RNA-sequencing from men patients. Differential analysis was carried out with ‘limma-voom’ (v.3.28.14) with the protein-coding genes. Statistical significance of genes was determined by an adjusted P-value according to the Benjamini-Hochberg procedure of p < 0.05.

**Supplementary Table 23.** The most differentially expressed hepatic progenitor (HB) genes between LPO (compared to control) and LPOT (compared to control) (|log2FC(LPO)-(log2FC(LPOT)| ≥ 0.5, adjusted P-value < 0.05 according to Bonferroni correction) from male liver spheres.

**Supplementary Table 24.** The most differentially expressed genes from male sphere hepatic progenitor (HB) cluster were compared to SteatoSITE bulk RNA-sequencing from men patients. Differential analysis was carried out with ‘limma-voom’ (v.3.28.14) with the protein-coding genes. Statistical significance of genes was determined by an adjusted P-value according to the Benjamini-Hochberg procedure of p < 0.05.

**Supplementary Table 25.** The most differentially expressed mesodermal progenitor genes between LPO (compared to control) and LPOT (compared to control) (|log2FC(LPO)-(log2FC(LPOT)| ≥ 0.5, adjusted P-value < 0.05 according to Bonferroni correction) from male liver spheres.

**Supplementary Table 26.** The most differentially expressed genes from male sphere mesodermal progenitor cluster were compared to SteatoSITE bulk RNA-sequencing from men patients. Differential analysis was carried out with ‘limma-voom’ (v.3.28.14) with the protein-coding genes. Statistical significance of genes was determined by an adjusted P-value according to the Benjamini-Hochberg procedure of p < 0.05.

**Supplementary Table 27.** The most differentially expressed mesenchymal stromal cell (MSC) genes between LPO (compared to control) and LPOT (compared to control) (|log2FC(LPO)-(log2FC(LPOT)| ≥ 0.5, adjusted P-value < 0.05 according to Bonferroni correction) from male liver spheres.

**Supplementary Table 28.** The most differentially expressed genes from male sphere mesenchymal stromal cell (MSC) cluster were compared to SteatoSITE bulk RNA-sequencing from men patients. Differential analysis was carried out with ‘limma-voom’ (v.3.28.14) with the protein-coding genes. Statistical significance of genes was determined by an adjusted P-value according to the Benjamini-Hochberg procedure of p < 0.05.

**Supplementary Table 29.** The most differentially expressed quiescent hepatic stellate cell (qHSC) genes between LPO (compared to control) and LPOT (compared to control) (|log2FC(LPO)-(log2FC(LPOT)| ≥ 0.5, adjusted P-value < 0.05 according to Bonferroni correction) from male liver spheres.

**Supplementary Table 30.** The most differentially expressed genes from male sphere quiescent hepatic stellate cell (qHSC) cluster were compared to SteatoSITE bulk RNA-sequencing from men patients. Differential analysis was carried out with ‘limma-voom’ (v.3.28.14) with the protein-coding genes. Statistical significance of genes was determined by an adjusted P-value according to the Benjamini-Hochberg procedure of p < 0.05.
